# PMEPA1 isoform a drives progression of glioblastoma by promoting protein degradation of the Hippo pathway kinase LATS1

**DOI:** 10.1038/s41388-019-1050-9

**Published:** 2019-10-11

**Authors:** Jianxiong Ji, Kaikai Ding, Tao Luo, Ran Xu, Xin Zhang, Bin Huang, Anjing Chen, Di Zhang, Hrvoje Miletic, Rolf Bjerkvig, Frits Thorsen, Jian Wang, Xingang Li

**Affiliations:** 1 Department of Neurosurgery, Qilu Hospital of Shandong University and Institute of Brain and Brain-Inspired Science, Shandong University, Jinan, China; 2 Shandong Key Laboratory of Brain Function Remodeling, Jinan, China; 3 School of Medicine, Shandong University, Jinan, China; 4 Department of Biomedicine, University of Bergen, Jonas Lies vei 91, 5009 Bergen, Norway; 5 Department of Pathology, Haukeland University Hospital, Jonas Lies vei 65, 5021 Bergen, Norway; 6 Department of Oncology, Luxembourg Institute of Health, 84, Val Fleuri, Luxembourg, L-1526 Luxembourg; 7 The Molecular Imaging Center, Department of Biomedicine, University of Bergen, Jonas Lies vei 91, 5009 Bergen, Norway

**Keywords:** CNS cancer, Ubiquitylation, Diagnostic markers

## Abstract

The Hippo signaling pathway controls organ development and is also known, in cancer, to have a tumor suppressing role. Within the Hippo pathway, we here demonstrate, in human gliomas, a functional interaction of a transmembrane protein, prostate transmembrane protein, androgen induced 1 (PMEPA1) with large tumor suppressor kinase 1 (LATS1). We show that PMEPA1 is upregulated in primary human gliomas. The PMEPA1 isoform PMEPA1a was predominantly expressed in glioma specimens and cell lines, and ectopic expression of the protein promoted glioma growth and invasion in vitro and in an orthotopic xenograft model in nude mice. In co-immunoprecipitation experiments, PMEPA1a associated with the Hippo tumor suppressor kinase LATS1. This interaction led to a proteasomal degradation of LATS1 through recruitment of the ubiquitin ligase, neural precursor cell expressed, developmentally downregulated 4 (NEDD4), which led to silencing of Hippo signaling. Alanine substitution in PMEPA1a at PY motifs resulted in failed LATS1 degradation. Targeting of a downstream component in the Hippo signaling pathway, YAP, with shRNA, interfered with the growth promoting activities of PMEPA1a in vitro and in vivo. In conclusion, the presented work shows that PMEPA1a contributes to glioma progression by a dysregulation of the Hippo signaling pathway and thus represents a promising target for the treatment of gliomas.

## Introduction

Dysregulation of the Hippo signaling pathway represents a common event in many cancers including glioma [1–4]. In recent years, large tumor suppressor kinase 1/2 (LATS1/2) and core components of Hippo signaling have received significant attention in human cancer. Once stimulated by upstream regulators, LATS1/2 phosphorylates YAP, leading to its cytoplasmic retention and inactivation; otherwise, YAP translocates to the nucleus and activates transcription of downstream proproliferative and antiapoptotic genes [5, 6]. LATS1 has been identified as a tumor suppressor, and decreased expression of LATS1 has been correlated with poor prognosis in glioma patients [7].

In a previous study, we demonstrated that LATS1 was downregulated in astrocytoma due to promoter hypermethylation, and that restoration of expression induced apoptosis in glioma cells [8]. More recent studies have shown that key components of Hippo signaling could be regulated by ubiquitination. A series of E3 ubiquitin ligases has been found to regulate several of these proteins, including LATS1 [9–11], LATS2 [12, 13], AMOT [13, 14], and YAP/TAZ [15, 16]. The E3 ubiquitin ligase neural precursor cell expressed, developmentally downregulated 4 (NEDD4), which recognizes a specific motif in proteins, the PY motif, leads to ubiquitination of LATS1/2 [11, 13]. Our recent work also illuminated a critical role for Hippo-YAP in the development of human glioma, which might involve regulation by proteasomal degradation [17].

Prostate transmembrane (TM) protein, androgen induced 1 (PMEPA1), also known as TM prostate androgen-induced RNA, reported to be induced by testosterone or its derivatives, has been shown to have a role in tumorigenesis [18, 19]. Additional studies have shown that epidermal growth factor, transforming growth factor-β (TGF-β), and mutant p53 also facilitate transcription of *PMEPA1* [20–22]. PMEPA1 is a type Ib TM protein containing two PY motifs that interact with HECT-type E3 ubiquitin ligases, such as NEDD4 [19]. Previous studies demonstrated that PMEPA1 is highly expressed in many solid tumor types, such as breast [23], prostate [18], lung [24], and ovarian cancers [20], but that it is difficult to detect in leukemias and lymphomas [25]. A number of studies have shown that PMEPA1 induces degradation of several proteins critical to the development of cancer, such as androgen receptor [26], TGF-β type I receptor [24], Smad 2/3 proteins [27], and c-Maf [28]. Thus, PMEPA1 could potentially act as a tumor suppressor gene or an oncogene.

Based on this prior knowledge, our aim was to unravel the underlying mechanisms of PMEPA1 function in human glioma progression. In this study, we show that the PMEPA1 protein is overexpressed in primary human glioma tissues and cell lines relative to nonneoplastic brain tissue samples and normal human astrocytes (NHA), where PMEPA1a is the predominant isoform in glioma samples and cell lines. The protein displayed a growth promoting activity in vitro and in vivo, and was found to interact directly with components of the tumor suppressing Hippo signaling pathway. Our results identify a role of PMEPA1a in the dysregulation of Hippo signaling and as a putative molecular target in the treatment of human glioblastomas (GBMs).

## Results

### PMEPA1 protein is overexpressed in human gliomas

**Fig. 1:**
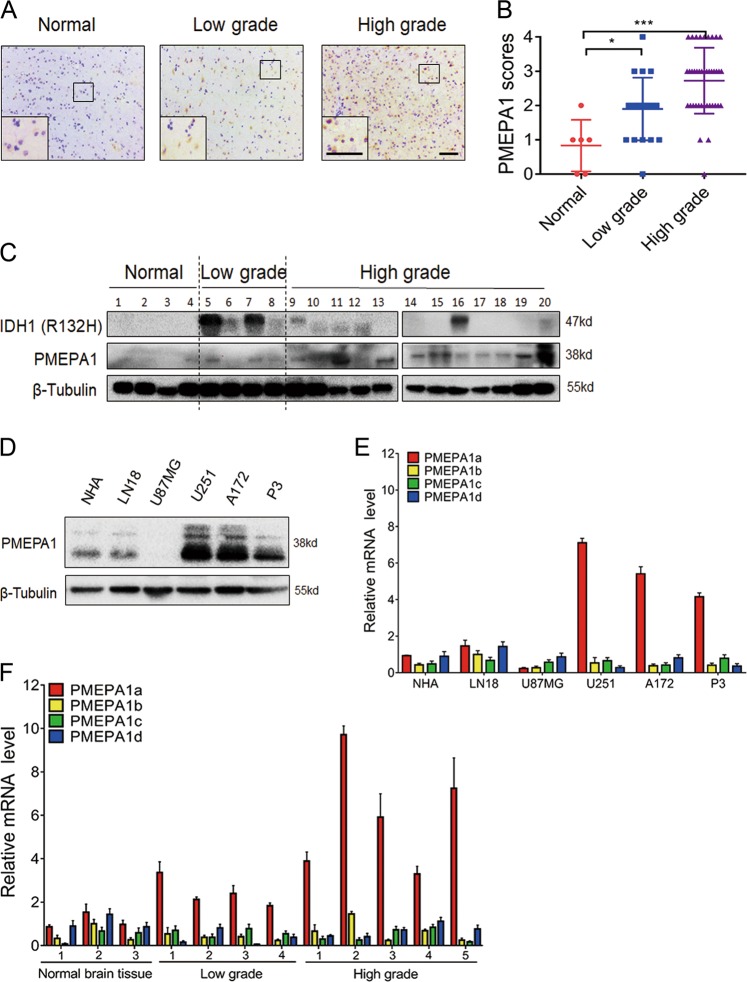
The levels of PMEPA1 protein and isoform are upregulated in primary glioma samples and cell lines. **a** Immunohistochemical staining for PMEPA1 in human glioma and nonneoplastic brain tissue samples. Scale bars, 100 µm. **b** Bar graphs indicate scoring performed on immunohistochemical staining for PMEPA1 across gliomas, both low grade and high grade, and nonneoplastic brain tissue samples. Data are represented as the mean ± SEM. **c** Western blot analysis of PMEPA1 protein levels in primary glioma tissue samples. **d** Western blot analysis of PMEPA1 protein levels in normal human astrocytes (NHA) and glioma cell lines. **e** qRT-PCR analysis of PMEPA1 isoforms in glioma cell lines. Relative expression levels based on normalization with GAPDH is plotted. PMEPA1a, PMEPA1b, PMEPA1c, and PMEPA1d are analyzed in NHA, LN18, U87MG, A172, U251, and P3 cell lines using isoform-specific primers. GAPDH was used for normalization. **f** qRT-PCR performed with isoform-specific primers on RNA from primary human glioma (*n* = 9) and nonneoplastic brain tissue samples (*n* *=* 3). Relative expression levels of PMEPA1a, PMEPA1b, PMEPA1c, and PMEPA1d were determined using GAPDH for normalization and plotted. Student’s *t*-test: *P *<* 0.05, ***P *<* 0.001

We found that PMEPA1 protein levels were increased in high grade gliomas (WHO III–IV; *n* = 40) relative to normal brain tissues (*n* = 6) and low grade gliomas (WHO II; *n* = 20; Fig. 1a, b). Western blotting of lysates prepared from primary tumors (*n* = 16; WHO grades II–IV) and nonneoplastic brain tissue samples (*n* = 4) also confirmed PMEPA1 overexpression in human gliomas (Fig. 1c), but no significant correlation with *IDH1* status. High expression (IHC score > 2) was observed in 4 of 20 low grade gliomas (WHO II; 20%), and 24 of 40 high grade gliomas (WHO III–IV; 60%), and was therefore significantly correlated with increasing tumor grade (Supplementary Table S1, *P* = 0.0034). PMEPA1 protein levels were also increased in glioma cell lines relative to NHA in culture, except in the case of U87MG, where PMEPA1 were nearly undetectable (NHA; Fig. 1d).

### *PMEPA1a* is more highly expressed in glioma tissues and cell lines than other alternatively spliced PMEPA1 isoforms

Four alternatively spliced isoforms exist for the *PMEPA1* gene (isoforms *PMEPA1a-d*), and the membrane-bound proteins (isoforms *PMEPA1a, b, d*) have been shown to exhibit oncogenic functions in prostate cancer cells and solid tumors, while cytosolic protein (isoform c) has no effect on cancer progression [27, 29]. We therefore evaluated expression levels of the *PMEPA1* isoforms in our glioma cell lines, using PCR primers specific for *PMEPA1a*, *PMEPA1b*, *PMEPA1c*, and *PMEPA1d* transcripts. *PMEPA1a* was the most highly expressed isoform, with a relative expression level > 5× higher than the other isoforms in U251, A172, and GBM#P3 cells. In U87MG cells, the *PMEPA1d* isoform was more highly expressed than others although overall relative levels were still quite low (Fig. 1e). We also assessed expression levels of *PMEPA1* isoforms in a cohort of primary glioma and nonneoplastic brain tissue samples. The relative levels of *PMEPA1a* in the tumor samples (*n* = 9) was higher than in normal brain tissues (*n* = 3; Fig. 1f). *PMEPA1a* may therefore be the isoform with the most significant role in glioma progression.

### PMEPA1a promotes glioma cell growth, migration, and invasion both in vitro and in vivo

We first examined the efficiency of our constructs for *PMEPA1a* shRNAs and ectopic expression of the various isoforms. We used two shRNAs to target PMEPA1a, and both led to a ~4× decrease in protein and mRNA levels as assessed by western blots and qRT-PCR in A172 and U251. Constructs for isoform PMEPA1a was efficiently expressed in U87MG cells (Supplementary Fig. S1A, B).

**Fig. 2:**
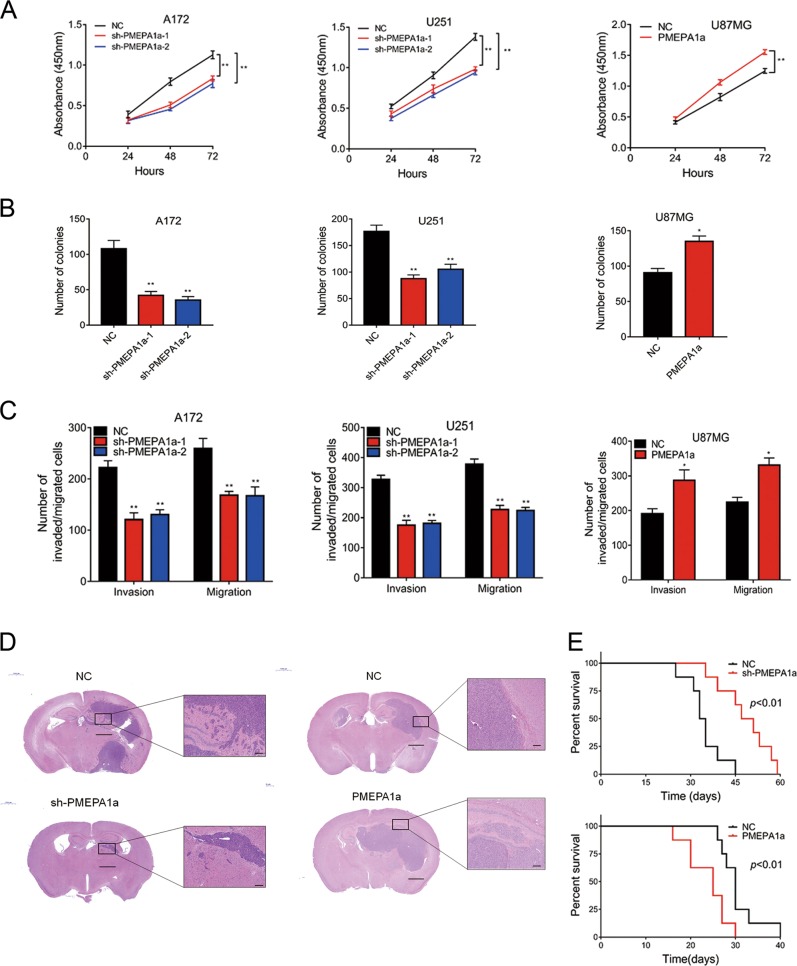
PMEPA1a promotes proliferation, migration, and invasion of glioma cells in vitro and in vivo. Cells were examined in **a** by CCK-8 and in **b** by colony forming assays. Data are represented as the mean ± SEM. **c** Graphic analysis of results from transwell assays performed on the indicated cells. Data are represented as the mean ± SEM from three independent experiments. **d** Representative images of hematoxylin and eosin-stained sections from the brains of nude mice implanted intracranially with modified U251 cells or U87MG cells. Scale bars, 100 µm, 1000 µm. **e** Kaplan–Meier survival analysis performed with survival data of mice implanted with indicated cells. Log-rank test, *P* *<* 0.01. Student’s *t*-test: n.s. = not significant, *P < 0.05, **P < 0.01

Growth was significantly decreased in A172- and U251-sh-PMEPA1a cells, but enhanced in U87MG-PMEPA1a cells (Fig. 2a). The results were corroborated in colony forming assays (Fig. 2b; Supplementary Fig. S2A); the number of colonies was reduced by ~50% in A172- and U251-sh-PMEPA1a cells, but increased ~30% in U87MG-PMEPA1a cells. Finally, migration and invasion were decreased in A172- and U251-sh-PMEPA1a cells, but enhanced in U87MG-PMEPA1a (~30%; Fig. 2c and Supplementary Fig. S2B).

Modified cells were also orthotopically implanted into the brains of nude mice to assess in vivo growth. HE staining revealed that U251-sh-PMEPA1a xenografts were more circumscribed than controls, whereas U87MG-PMEPA1a cells were more invasive (Fig. 2d). Immunohistochemical staining of sections also highlighted differences in growth characteristics between xenografts. Ki67, a marker for proliferation, was positively correlated with PMEPA1a expression levels, whereas LATS1 was negatively correlated with PMEPA1a (Supplementary Fig. S2C, D). Finally, overall survival of animals was enhanced by PEMPA1a knockdown (median survival, 34 vs. 49 days, U251-NC and U251-sh-PMEPA1a, respectively, *P* < 0.01; Fig. 2e), but decreased in tumor bearing animals from the U87MG-PMEPA1a group (median survival, 30 vs. 25 days, U87MG-NC and U87MG-PMEPA1a, respectively, *P* < 0.01; Fig. 2e). All together, these results indicated that PMEPA1a promoted glioma progression in vivo.

### LATS1 is a PMEPA1a-interacting protein

To delineate the pathway(s) involved in the PMEPA1a response, we performed co-immunoprecipitation (Co-IP) assays to identify partners of the protein in human gliomas. U87MG cells were transfected with Flag-tagged PMEPA1a or control vector (Flag-empty-vector), and Co-IP assays were performed followed by proteomic analysis of the isolated PMEPA1a-associated protein complexes (Supplementary Fig. S3A, B). Mass spectrometry revealed known PMEPA1a-interacting proteins, such as NEDD4, but also some novel proteins. According to TCGA database, MYH9, DHX9, DDB1, and HMGB1 do not show distinct expression levels between nonneoplastic and GBM samples (Data not shown here), while VDAC1 had been reported to play a role in GBM cell metabolism and MTA2 expression level was related to glioma cells proliferation and invasion [30, 31]. Interestingly, LATS1, a key component of the Hippo pathway, was also one of the top interacting proteins. According to our previous study, Hippo signaling pathway is of vital importance to maintain malignant behaviors of glioma cells and worthy investigating further [17]. Therefore, we select LATS1 kinase as our main point in the mechanical study.

**Fig. 3:**
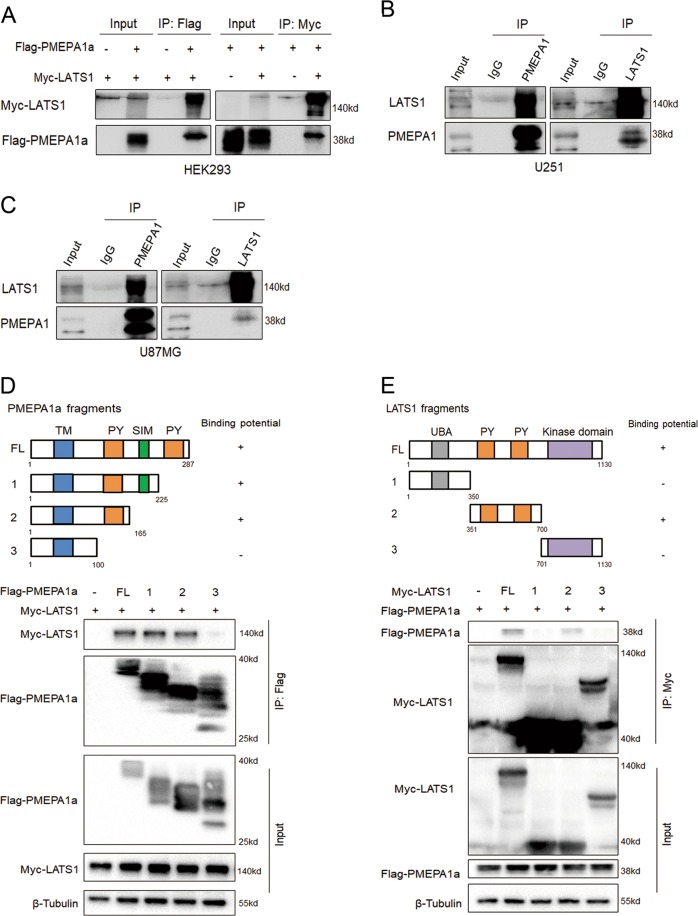
PMEPA1a physically interacts with LATS1. Co-immunoprecipitations performed and analyzed in **a**–**c** western blotting to detect interaction between PMEPA1a and LATS1 in HEK293 cells transfected with Myc-tagged LATS1 and Flag-tagged PMEPA1a, and in parental U87MG and U251 cells. **d** Schematic representation of wild-type PMEPA1a and the indicated deletion mutants. Western blot analysis of co-immunoprecipitations performed on lysates prepared from HEK293 cells transfected with Myc-LATS1 alone or together with indicated Flag-PMEPA1a constructs. Upper panels represent Co-IP performed with anti-Flag; lower panels represent total protein. **e** Schematic representation of wild-type LATS1 and the indicated deletion mutants. Western blot analysis of co-immunoprecipitations performed on lysates prepared from HEK293 cells transfected with Flag-PMEPA1a alone or together with indicated Myc-LATS1 constructs. Upper panels represent Co-IPs performed with anti-MYC; lower panels represent total protein. Experiments were performed in triplicate

To verify the interaction between PMEPA1a and the LATS1 kinase, we simultaneously expressed Flag-tagged PMEPA1a and Myc-tagged LATS1 in HEK293 cells. In these cells, we could confirm an interaction between Flag-PMEPA1a and Myc-LATS1, where the Co-IP was performed with the Flag or the Myc antibody (Fig. 3a). We also confirmed the existence of endogenous complexes containing PMEPA1 and LATS1 in U251 and U87MG cells (Fig. 3b, c). Complexes brought down with either PMEPA1 or LATS1 antibody contained both proteins.

To map the binding region for LATS1 and PMEPA1a, deletion mutants of LATS1 and PMEPA1a were constructed and expressed in cells. The results indicated that amino acids 100–165 in PMEPA1a were necessary for binding to LATS1, whereas amino acids 351–700 in LATS1 constituted the PMEPA1a-interacting domain (Fig. 3d, e).

### PMEPA1a regulates the Hippo kinase signaling pathway

**Fig. 4:**
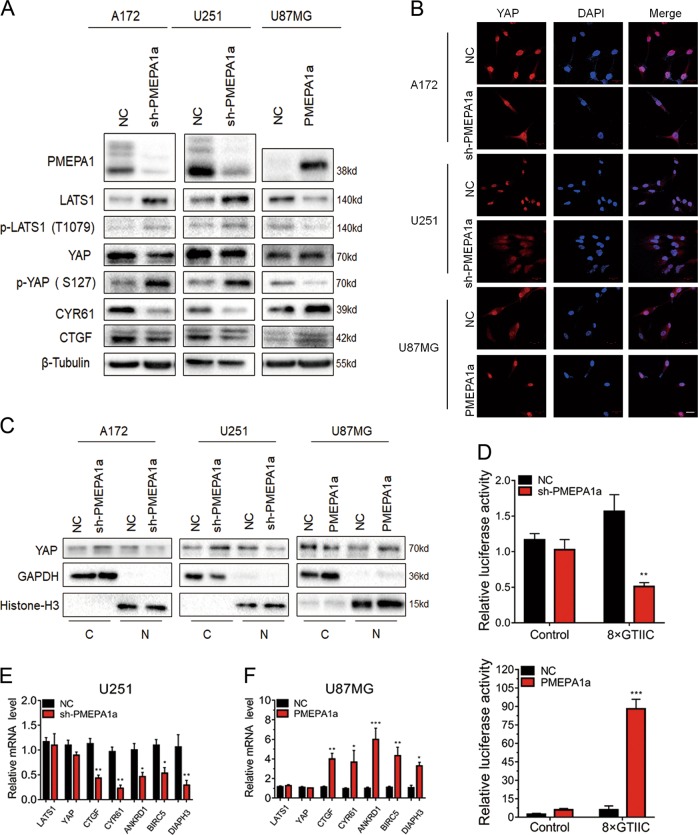
PMEPA1a suppresses the Hippo kinase signaling through LATS1. **a** Western blot analysis to evaluate components in the Hippo kinase pathway downstream of PMEPA1a in lysates prepared from glioma cell lines, which were modified with PMEPA1a or sh-PMEPA1a as indicated. β-Tubulin was used as loading control. **b** Representative images of immunofluorescence staining for YAP (red) in modified glioma cell lines showing cellular localization. Nuclei are stained with DAPI (blue). Scale bars, 20 µm. **c** Western blot analysis of cytoplasmic (C) and nuclear (N) fractions prepared from indicated cells. **d** Luciferase assay for 8xGTIIC-Lux or control reporter constructs indicating YAP-dependent transcriptional activity in modified U87MG and U251 cells. Data are normalized to a Renilla reporter and to the NC group. **e**, **f** qRT-PCR analysis of modified U87MG and U251 cells. GAPDH was used as loading control. Data are normalized to the NC group. Student’s *t*-test: *P *<* 0.05, **P *<* 0.01, ***P *<* 0.001

LATS1 is a tumor suppressor in the Hippo signaling pathway and inactivates the oncogenic transcriptional regulator YAP through phosphorylation at ser-127 [1, 15]. Loss of LATS1 can lead to a corresponding increase in YAP activity. Western blot analysis revealed decreased LATS1 protein levels in U87MG-PMEPA1a cells. In contrast, LATS1 was increased in A172- and U251-sh-PMEPA1a cells (Fig. 4a). Thus, PMEPA1a suppressed LATS1 protein in glioma cells with an endogenous PMEPA1a expression.

Furthermore, altered PMEPA1a also led to detectable changes in YAP and its activity. Nuclear localization of YAP was enhanced in U87MG-PMEPA1a cells and decreased in A172- and U251-sh-PMEPA1a cells (Fig. 4b, c). These results are consistent with an activation of YAP through increased PMEPA1a. Indeed, analysis of the transcriptional activity of YAP supported this hypothesis. We demonstrated increased YAP activity in the presence of PMEPA1a through a YAP promoter reporter luciferase construct and qRT-PCR of YAP endogenous downstream genes (Fig. 4d–f). These data together demonstrate that PMEPA1a increases YAP activity by decreasing LATS1 protein levels.

### PMEPA1a promotes proteasomal degradation of LATS1

**Fig. 5:**
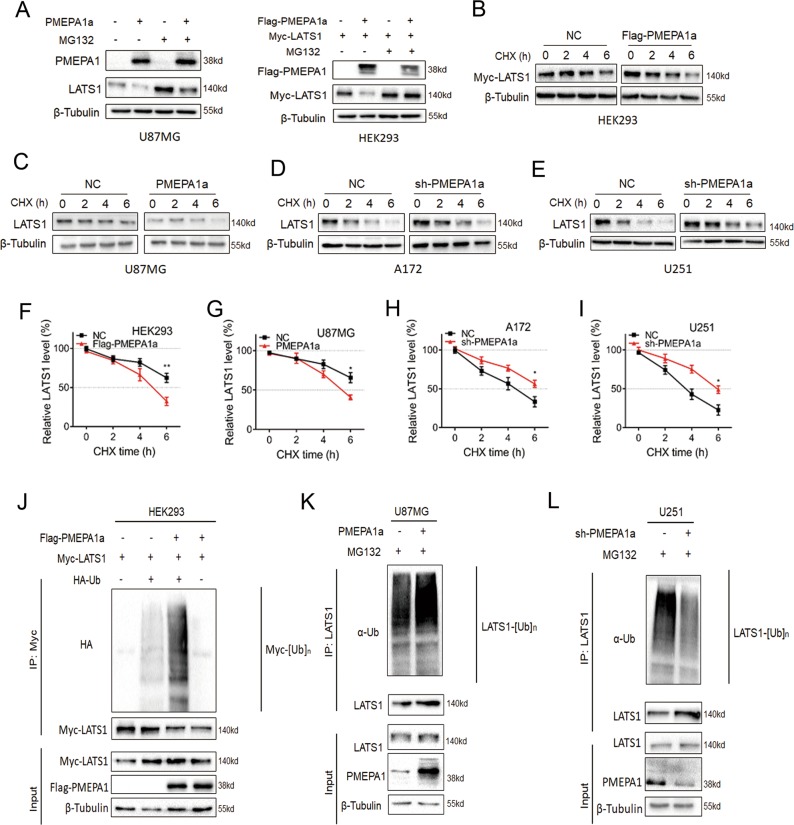
PMEPA1a destabilizes LATS1 proteins. **a** Western blot analysis to evaluate LATS1 levels in U87MG- and HEK293-NC and -PMEPA1a cells after treatment with proteasome inhibitor MG132 (20 µM) for 8 h. β-tubulin was used as the loading control. **b**–**e** Western blot analysis of LATS1 protein in modified HEK29, U87MG, A172, and U251 cells treated with cycloheximide (CHX; 25 µg/mL) for the indicated time. (**f**–**i**) Line graphs representing LATS1 levels normalized to β-tubulin and to 0 h at the indicated time points from CHX experiments (*n* = 4). Data are represented as the mean ± SEM. (**j**–**l**) Western blot analysis of ubiquitination assays. Cells were transfected with PMEPA1a in HEK293 and U87MG or shRNA (sh-PMPA1a) in U251 cells. Cells were treated with MG132 (20 µM) for 8 h. Student’s *t*-test: *P *<* 0.05, **P *<* 0.01

Altered expression of PMEPA1a had no obvious effects on mRNA levels of *LATS1* (Fig. 4e, f). Therefore, we tested whether PMEPA1a modulates LATS1 protein stability. We first examined LATS1 protein levels in modified cell lines treated with the proteasome inhibitor MG132. We found that MG132 partially reversed the downregulation of LATS1 protein in U87MG- and HEK293-PMEPA1a cells (Fig. 5a). In addition, the half-life of LATS1 protein was altered in these cells. In the presence of the protein synthesis inhibitor cycloheximide (CHX), we found the half-life of LATS1 to be reduced in U87MG- and HEK293-PMEPA1a cells (Fig. 5b, c, f, g). In contrast, the half-life of LATS1 protein was prolonged in A172- and U251-sh-PMEPA1a cells (Fig. 5d, e, h, i). Thus, PMEPA1a promotes proteasome-mediated degradation of LATS1.

Levels of LATS1 ubiquitination were also consistent with increased proteasomal degradation. In HEK293-Flag-PMEPA1a cells expressing HA-Ub, ubiquitination of LATS1 was increased (Fig. 5J). Furthermore, endogenous ubiquitination of LATS1 was regulated through PMEPA1a. In U251-sh-PMEPA1a cells, the polyubiquitination of LATS1 was reduced, while in U87MG-PMEPA1a cells, the polyubiquitination of LATS1 was increased (Fig. 5k, l). Taken together, PMEPA1a plays a critical role in regulating the protein levels of LATS1 mediated by proteasomal degradation.

### PMEPA1a destabilizes LATS1 through the E3 ligase NEDD4

**Fig. 6:**
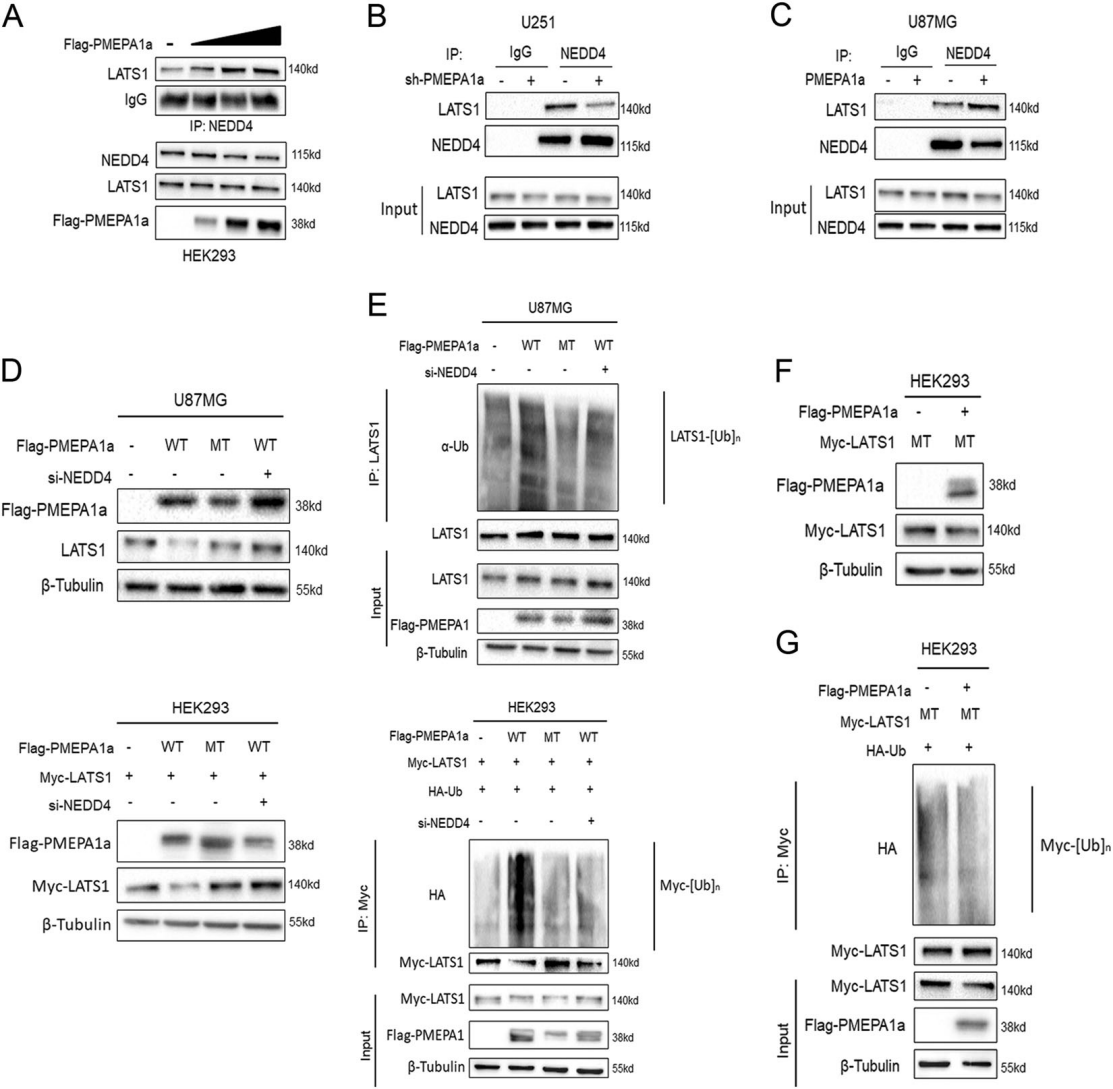
PMEPA1a promotes ubiquitination of LATS1 by facilitating its interaction with NEDD4. **a** Western blot analysis of co-immunoprecipitations performed with anti-NEDD4 and lysates prepared from HEK293 cells transfected with increasing amounts of Flag-PMEPA1a. Top panel corresponds to western blot for co-IPs; bottom panel corresponds to total protein in lysates. Cells were pretreated with MG132 (20 µM) for 8 h. **b**, **c** Western blot analysis of co-immunoprecipitations performed with anti-NEDD4 and lysates prepared from modified U87MG and U251 cells. Cells were pretreated with MG132 (20 µM) for 8 h. **d** Western blot analysis of lysates prepared from cells 48 h after transfection as indicated. U87MG cells were transfected with Flag-PMEPA1a wild type (WT) alone, Flag-PMEPA1a mutated (MT) alone, or with siNEDD4 as indicated. HEK293 cells were transfected with Myc-LATS1, Flag-PMEPA1a (WT) alone, Flag-PMEPA1a (MT) alone, or with siNEDD4 as indicated. **e** Western blot analysis of ubiquitination assays. U87MG cells were transfected with Flag-PMEPA1a (WT) alone, Flag-PMEPA1a (MT) alone, or with siNEDD4. HEK293 cells were transfected with Myc-LATS1, Flag-PMEPA1a (WT) alone, Flag-PMEPA1a (MT) alone, or with siNEDD4. **f** Western blot analysis of total protein in lysates prepared from HEK293 cells transfected with Myc-LATS1 (MT) alone, or together with Flag-PMEPA1a (WT). **g** Western blot analysis of ubiquitination assays. Lysates were prepared from HEK293 cells transfected with Myc-LATS1 (MT) alone, or together with Flag-PMEPA1a (WT)

Previous studies have demonstrated that PMEPA1 mediates protein degradation through its natural protein partner, NEDD4 [26, 28]. As LATS1 was recently identified as a novel substrate of NEDD4 [11, 13], we therefore performed Co-IPs to determine the relationship between these three proteins. HEK293 cells were transfected with increasing levels of Flag-PMEPA1a, and Co-IPs were performed on lysates with anti-NEDD4 antibodies. Under these conditions, LATS1 complexed with NEDD4 was increased (Fig. 6a). Co-IPs were also performed with lysates prepared from U251-sh-PMEPA1a and U87MG-PMEPA1a cells. In the absence of PMEPA1a in U251-sh-PMEPA1a, the amount of LATS1 complexed with NEDD4 was decreased (Fig. 6b). However, with increased expression of PMEPA1a in U87MG cells, the amount of LATS1 complexed with NEDD4 was increased (Fig. 6c). Thus, an increase or a decrease in the levels of PMEPA1a produced a corresponding increase or decrease in the amount of LATS1 in complex with NEDD4.

To further confirm that PMEPA1a regulates the level of LATS1 through NEDD4-mediated proteasomal degradation, the PMEPA1a mutant (MT; Y161/232A), which exhibits nearly undetectable binding to NEDD4, was transfected into U87MG and HEK293 cells (Supplementary Fig. S4A). On western blot, levels of LATS1 did not decrease in the presence of PMEPA1a MT (Fig. 6d) even though this mutant retained LATS1-binding ability (Supplementary Fig. S5A, B). Ubiquitination of LATS1 was also not increased in the presence of transfected PMEPA1a MT (Fig. 6e). Alternatively, knockdown of NEDD4 with a small interfering RNA (si-NEDD-2; Supplementary Fig. S6A–C) produced similar results; wild-type PMEPA1a failed to lead to reduced LATS1 protein levels (Fig. 6d) or increased polyubiquitination (Fig. 6e). Two constructs for PMEPA1a mutants, PMEPA1a-Y161/232A and PMEPA1a-Δ100-165aa, were efficiently expressed in U87MG cells (Supplementary Fig. S7A). Cell growth was markedly enhanced in U87MG-PMEPA1a cells, but remained unchanged in U87MG-PMEPA1a-Y161/232A or PMEPA1a-Δ100-165aa cells compared with NC group (Supplementary Fig. S7B). The results were corroborated in colony forming assays and transwell assays (Supplementary Fig. S7C, D). luciferase-expressing modified U87MG cells were orthotopically implanted into the brains of nude mice to assess in vivo growth. In vivo bioluminescence revealed that ectopic expression of PMEPA1a in U87MG cells led to increased cell growth in vivo and reduced the survival time of tumor bearing mice (~25 vs. 30 days, PMEPA1a vs. NC*, P* < 0.05), while PMEPA1a-Y161/232A or PMEPA1a-Δ100-165aa had no effect on cell growth, and overall survival remained unchanged in these two groups compared with NC group either (33 or 30 vs. 30 days, PMEPA1a-Y161/232A or PMEPA1a-Δ100-165aa vs. NC, *P* = n.s.; Supplementary Fig. S7E–G).

To further validate this mechanism, a LATS1 mutant (LATS1 MT; Y376/559A), which is insensitive to NEDD4-mediated proteasomal degradation, was transfected into HEK293 cells along with PMEPA1a (Supplementary Fig. S4B). We found that although LATS1 MT was still able to associate with PMEPA1a (Supplementary Fig. S5A, B), LATS1 MT protein levels remained unchanged (Fig. 6f). Ectopic expression of *PMEPA1a* also did not result in increased polyubiquitination of LATS1 MT, which further corroborated the absence of an interaction with NEDD4 (Fig. 6g).

These results suggested that the interaction between PMEPA1a and NEDD4 was essential for regulating LATS1. Moreover, the effect of PMEPA1a on LATS1 protein could be abrogated by deletion of NEDD4.

### The Hippo pathway mediates PMEPA1a signaling

To examine whether the Hippo pathway is downstream of PMEPA1a, we manipulated LATS1 and YAP levels using shRNAs and expression constructs in U251-sh-PMEPA1a or U87MG-PMEPA1a cells (Supplementary Fig. S8A) and assessed characteristics in growth curves, colony forming assays, and migration assays. The expression construct for YAP contained a constitutively active form of the protein due to mutations in the inhibitory LATS1/2 phosphorylation sites [15]. Growth was inhibited in U251-sh-PMEPA1a cells relative to controls, but knockdown of LATS1 or overexpression of YAP (YAP-5SA) in these cells enhanced growth and migration (Supplementary Figs. S8B–D; S9A, B). However, in U87MG-PMEPA1a cells, lentivirus expressing LATS1 or shRNA targeting YAP reduced cell growth and migration (Supplementary Figs. S8B–D; S9A, B).

In a subcutaneous tumor model, the oncogenic effect of PMEPA1a on glioma growth was also shown to be mediated by Hippo signaling/YAP. Tumor growth of U251-sh-PMEPA1a cells increased with knockdown of LATS1 or overexpression of YAP (Supplementary Figs. S8E; S9C, D). In contrast, tumor growth of U87MG-PMEPA1a cells was decreased with overexpression of the tumor suppressor LATS1 or knockdown of YAP (Supplementary Figs. S8E; S9C, D). Collectively, these data demonstrated that the Hippo-LATS1-YAP axis might play a functional role in PMEPA1a-induced the development of human glioma.

**Fig. 7:**
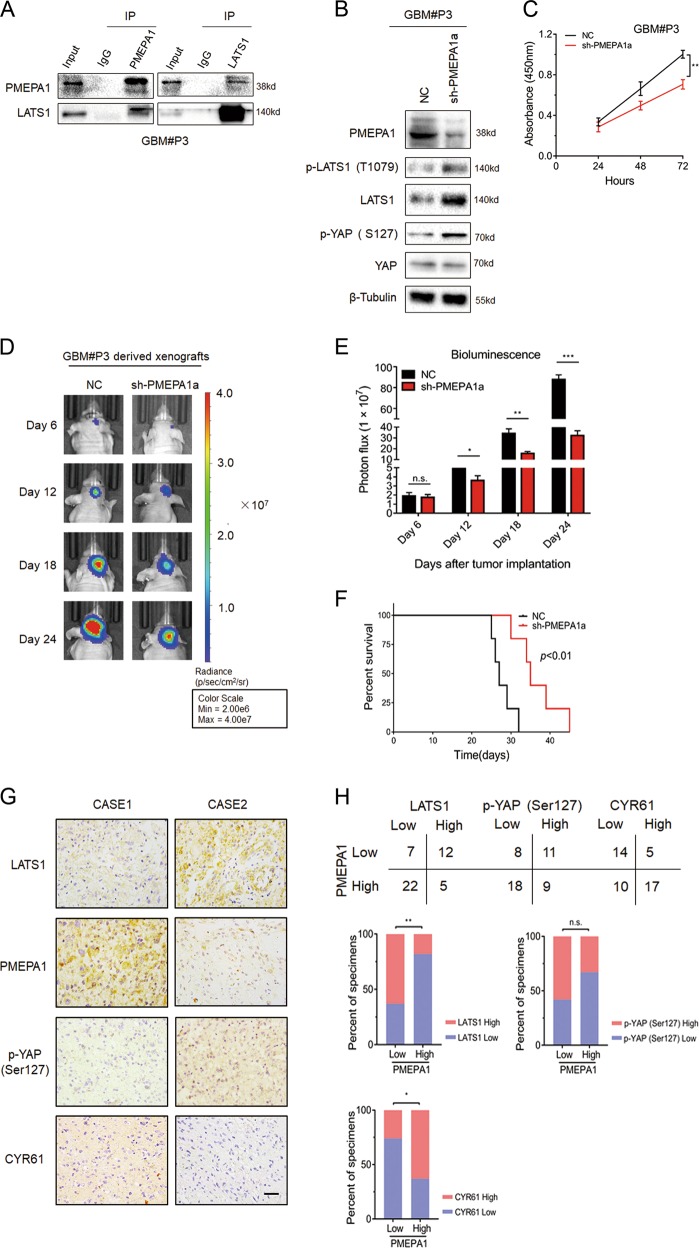
PMEPA1a knockdown inhibits glioma growth in GBM#P3 cells in vitro and in vivo. **a** Co-immunoprecipitations to demonstrate association of PMEPA1a with LATS1 in GBM#P3 cells. **b** Western blot analysis to evaluate components of the Hippo kinase pathway in lysates prepared from GBM#P3-NC and -sh-PMEPA1a cells. β-tubulin was used as loading control. **c** Absorbance values (450 nm) obtained from the CCK8 assay performed on GBM#P3-NC and -sh-PMEPA1a cells. Data are represented as the mean ± SEM. **d**, **e** In vivo bioluminescent images and quantification of GBM#P3-NC and -sh-PMEPA1a derived xenografts at the indicated time points. **f** Kaplan–Meier survival analysis performed with survival data from mice implanted with GBM#P3-NC and -sh-PMEPA1a cells. Log-rank test, *P* *<* 0.01. **g** Representative images of IHC staining of PMEPA1 and LATS1 in primary human glioma tissue samples. Scale bars, 50 μm. **h** Correlation analysis for PMEPA1 with LATS1 in primary human glioma samples based on IHC scoring. IHC scores are indicated in parentheses. *χ*^2^-test, *P* = 0.0020. Student’s *t*-test: n.s. = not significant, *P *<* 0.05, **P < 0.01, ***P < 0.001

Data from experiments on immortal GBM cell lines do not fully mimic the tumor heterogeneity observed in GBM patients. Thus, we performed additional experiments using GBM#P3 cells, which more faithfully retain the genetic features of the matching primary GBM [32]. In Co-IPs, PMEPA1 was also found to interact with LATS1 (Fig. 7a). In western blots, LATS1 protein levels increased in GBM#P3-sh-PMEPA1a cells as well as phosphorylated YAP (Fig. 7b). Knockdown of PMEPA1a in GBM#P3 cells also led to decreased cell growth in vitro (Fig. 7c) and in vivo (Fig. 7d, e), and prolonged the survival time of tumor bearing mice (~28 vs. 34 days, control vs. PMEPA1a knockdown; Fig. 7f).

Finally, in primary human glioma samples, IHC scores for PMEPA1 negatively correlated with scores for LATS1 (*P* *<* 0.01) and positively correlated with scores for CYR61 (*P* *<* 0.05), indicating a possible relationship between the proteins, while no significant correlation was found between PMEPA1 and p-YAP (Ser127) (Fig. 7g, h). These results are in agreement with our proposed mechanism for PMEPA1a, which could downregulate the protein levels of LATS1 and then play an oncogenic role in human gliomas.

## Discussion

Here, we identified the functional roles of the PMEPA1a isoform in glioma progression and a pathway mediating its activity. Our data show that the PMEPA1a isoform is highly expressed in human gliomas, and overexpression of the protein enhanced growth characteristics of glioma cell lines in vitro and in vivo. PMEPA1a promotes LATS1 ubiquitination and degradation by recruiting the E3 ligase NEDD4, which leads to inhibition of Hippo signaling and activation of the growth promoting gene YAP. PMEPA1a therefore has a putative oncogenic role in human gliomas.

Previous studies have already revealed the different roles of PMEPA1 in various cancers [18–25]. In our study, we performed both gain- and loss-of-function experiments in vitro and in vivo to demonstrate that PMEPA1 plays an oncogenic role in glioma progression. We found that PMEPA1a interferes with Hippo signaling by directly interacting with LATS1, which enables association with NEDD4 and subsequent proteasomal mediated degradation. LATS1 protein did not show being degraded when PY motifs of PMEPA1a were mutated or in the absence of NEDD4. These results corroborate previous studies reporting failure of LATS1 to interact with NEDD4 mutated at PY motifs and thus, decreased proteasomal degradation [11]. We found that PMEPA1a also failed to regulate LATS1 mutated at PY motifs. This finding is consistent with a previous study, in which PY-mutated or PY-deleted PMEPA1 loses its ability to promote c-Maf degradation [28]. These results are consistent with the formation of a PMEPA1a/NEDD4/LATS1 complex, which induces polyubiquitination and finally degradation of LATS1.

**Fig. 8:**
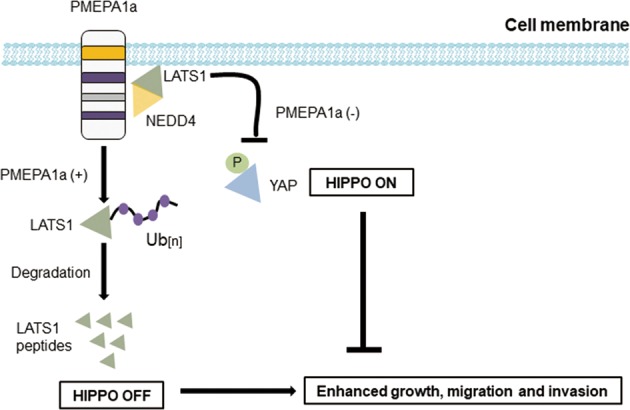
PMEPA1a promotion of tumor progression is mediated by Hippo signaling. PMEPA1a promotes tumor growth by facilitating degradation of LATS1, an important tumor suppressor protein inhibiting activity of YAP, a transcriptional activator of growth promoting genes. In cells overexpressing PMEPA1a, YAP is activated due to the loss of LATS1, while PMEPA1a depletion lead to increased YAP phosphorylation at ser-127 and foster cytoplasmic sequestration, causing inhibited phenotypes

However, how PMEPA1 isoform a had a significant oncogenic function in glioma is not clear. Potential explanation may be based on two critical factors. Firstly, the transmembrane (TM) domain is essential for PEMPA1 activities [27, 28] and secondly, NEDD4 is activated at the plasma membrane by phosphorylation or other factors [14, 33]. Based on our data, we propose the following model (Fig. 8). Membrane-bound PMEPA1, which possesses an N-terminal region, similar to isoform c, but also a TM domain, recruits NEDD4 to the inner side of the plasma membrane where it can be more easily activated. Activated NEDD4 induces LATS1 ubiquitination and degradation. We also found that amino acids 100–165 of PMEPA1a contain the PY motif closest to the TM domain, and may be responsible for the interaction between PMEPA1a and LATS1. We propose that membrane-bound PMEPA1 forms a complex with NEDD4 and LATS1 near the inner side of the plasma membrane, where activated NEDD4 interacts with LATS1, which results in polyubiquitination and finally degradation of LATS1. Further investigation is however required to confirm this hypothesis.

Many studies have demonstrated that Hippo signaling, which is often dysregulated and inactivated in many cancers, is critical for tumor progression [34, 35]. More recent reports revealed a set of E3 ubiquitin ligases, which destabilize key members of the Hippo pathway, illuminating another class of proteins with potential to drive tumorigenesis [9–16]. Recently, we have reported that actin-like 6A promotes YAP/TAZ protein stability by preventing interaction with beta-TrCP, which results in transcriptional activation of growth promoting genes underlying glioma progression [17]. In this study, we found that PMEPA1a regulates LATS1 protein by facilitating its interaction with NEDD4, an E3 ubiquitin ligase, and thus proteasomal degradation, which leads to nuclear accumulation and activation of YAP. Moreover, in PMEPA1a knockdown experiments, growth promoting activities of the transmembrane protein could be partially restored through manipulation of downstream components in the Hippo pathway, indicating that the Hippo pathway is likely to mediate the oncogenic functions of PMEPA1a in the development of human glioma.

In conclusion, our study demonstrates that membrane-bound PMEPA1, especially PMEPA1a, plays an oncogenic role in glioma progression. PMEPA1a facilitates association of LATS1 with NEDD4, which results in polyubiquitination and proteasomal degradation. Overexpression of PMEPA1a effectively leads to dysregulation of the Hippo pathway. Finally, this study identifies PMEPA1a as a putative molecular target for therapeutic strategies in the treatment of human gliomas.

## Materials and methods

### Ethics statement

All experiments and the use of human tissues were approved by the Research Ethics Committee of Shandong University (Shandong, China). Human brain tumor and nonneoplastic tissue samples were obtained from surgeries performed at the Department of Neurosurgery at Qilu Hospital (Shandong, China). Informed written consent was obtained from all patients. Normal brain tissues were obtained from brain trauma patients who underwent partial cerebral resection. All animal studies were approved by and performed under the guidance of the Institutional Animal Care and Use Committee (IACUC) of Shandong University.

### Cell culture and transfection

HEK293 cells and human glioma cell lines, LN18, U87MG, U251, and A172 were obtained from the Culture Collection of the Chinese Academy of Sciences (Shanghai, China). NHA and primary glioblastoma (GBM) #P3 cells were obtained from the University of Bergen, Norway. The plasmids used are listed in Supplementary Table S2. The sequences of shRNAs and siRNAs used are listed in Supplementary Table S3.

### Nuclear and cytoplasmatic fractionation

Nuclear and Cytoplasmic Extraction Reagents (Thermo Fisher Scientific lnc., Waltham, MA, USA) were used to obtain nuclear and cytoplasmic cellular subfractions according to the manufacturer’s instructions. Subcellular distribution of proteins, including YAP, were determined using western blot analysis. GAPDH and Histone H3 served as loading controls for cytosolic and nuclear protein fractions. See more details in Supplementary Methods and Materials. Experiments were performed in triplicate.

### Immunohistochemistry, immunofluorescence, and immunoblotting

Immunohistochemistry (IHC), immunofluorescence, and immunoblotting were performed as previously described [36]. Images were acquired using a Zeiss LSM780 confocal microscope (Carl Zeiss Microscopy GmbH, Jena, Germany). The IHC-stained samples were reviewed and evaluated by two blinded pathologists. Scores were determined on a scale of 0–4 by as follows: 0, no staining; 1, weak staining in <50% cells; 2, weak staining in ≥50% cells; 3, strong staining in <50% cells; and 4, strong staining in ≥50% cells. Immunoblotting experiments were performed in triplicate.

### Co-immunoprecipitation (Co-IP)

Cells were lysed in RIPA buffer (Pierce; Rockford, IL, USA) containing a protease inhibitor cocktail (Sigma). Total protein (200 µg; 1 µg/µL) was incubated with primary antibodies (4 µL) or IgG (4 µL) overnight at 4 °C with gentle shaking and then Protein A/G magnetic beads (Thermo Fisher Scientific) for 2 h at room temperature. The immunoprecipitated complexes were washed, boiled in protein loading buffer, and immunoblotted or subjected to mass spectrometry analysis (ekspertTMnanoLC; AB Sciex TripleTOF 5600-plus; SCIEX; Redwood City, CA, USA). Results of LC–MS/MSF were analyzed using ProteinpilotTM software (SCIEX). For the ubiquitination assay, cells were treated with 20 µM MG132 for 6 h before lysis, and subsequent co-IP and western blot analysis.

### Cycloheximide (CHX) chase

*LATS1* and *PMEPA1a* expression plasmids were cotransfected into HEK293 cells using Lipofectamine 3000 (Thermo Fisher Scientific). U87MG cells were infected with lentivirus for ectopic expression of full-length *PMEPA1a* (OBiO Technology). After 48 h, CHX **(**25 µg/mL**)** was added to the culture medium to inhibit translation, and cells were incubated for 0, 2, 4, or 6 h. Cell lysates were prepared, and protein (20 µg) was examined using western blot analysis. Experiments were performed in triplicate.

### Cell migration and invasion assay

Cell migration and invasion assays were performed as previously described [17]. Matrigel-coated (BD Biosciences, Bedford, MA, USA) and uncoated transwell chambers (pore size: 8 μm; Corning Costar, NY, USA) were used to evaluate cell invasion and migration accordingly. Experiments were performed in triplicate.

### Cell viability assay

Cell viability assay was performed as previously described [37]. After transfection for 48 h, cells were seeded into 96-well plates (5 × 10^3^ cells/well) and incubated at 37 °C overnight. After incubation with CCK-8 solution (10 μL/well; Dojindo; Kumamoto, Japan), the absorbance at 450 nm (OD450) was measured in a microplate reader (Bio-Rad; Hercules, CA, USA), and the results were plotted against time in days to generate growth curves. Experiments were performed in triplicate.

### Colony forming assay

After transfection, cells (1.0 × 10^3^/well) were seeded into six-well plates and cultured for an additional 2 weeks. Cells were fixed with 4% paraformaldehyde (Solarbio; Beijing, China) and stained with 5% crystal violet. Colonies of more than 50 cells were counted. Experiments were performed in triplicate.

### Reverse transcription PCR

Total RNA was isolated from cells or human tissues using Trizol Reagent (Thermo Fisher Scientific). RNA (2 µg) was reverse transcribed into cDNA using the High Efficient Reverse Transcription Kit (Toyobo Life Science; Shanghai, China) according to the manufacturer’s protocol. Quantitative PCR was performed using SYBR premix Ex Taq (Takara; Tokyo, Japan) on the Real-Time PCR Detection System (Roche, 480II; Basel, Switzerland). GAPDH served as the internal control for normalization. Primers used for PCR are listed in Supplementary Table S4. Experiments were performed in triplicate.

### Luciferase reporter assays

Modified U87MG and U251 cells were cotransfected with firefly luciferase (100 ng) and renilla reporters (100 ng) using Lipofectamine 3000 (Thermo Fisher Scientific). Luciferase assays were performed 24 h later using the Dual-Luciferase Reporter Assay Kit (Promega). Renilla activity was used to normalize luciferase reporter activity. Experiments were performed in triplicate.

### Animal studies

Stable transfected glioma cell populations (1 × 10^6^ cells) were implanted into the frontal lobes of 4-week-old nude mice (Shanghai SLAC Laboratory Animal Co., Ltd; Shanghai, China) using a stereotactic frame (KDS310, KD Scientific; Holliston, MA, USA).

Luciferase-expressing GBM#P3 and U87MG cells were implanted orthotopically in nude mice brains, and tumor growth was examined using bioluminescence (IVIS SPECTRUM, PerkinElmer; Hopkinton, MA, USA). The subcutaneous GBM model was established as previously described [17].

### Statistical analysis

Data are presented as mean ± SEM. The Student’s *t*-test for paired data was used to compare mean values. ANOVA was used to analyze potential differences between two groups with continuous variables. Survival curves were estimated by the Kaplan–Meier method and compared using the log-rank test. Correlation between *PMEPA1* and *LATS1*, *p-YAP (Ser127)*, or *CYR61* expression levels was determined using the two-tailed *χ*^2^ test or the Fisher’s exact test. Statistical analysis was conducted using GraphPad Prism version 7.00 software program for Windows (GraphPad; La Jolla, CA, USA). All tests were two-sided, and *P*-values < 0.05 were considered to be statistically significant.

## Supplementary information

Supplementary Figures and Materials and Tables
