# Supplementary material for: PMEPA1 isoform a drives progression of glioblastoma by promoting protein degradation of the Hippo pathway kinase LATS1
Source: Oncogene. 2019 Oct 11;39(5):1125–39. doi: 10.1038/s41388-019-1050-9 (PMC6989403; doi:10.1038/s41388-019-1050-9)
Supplement: Supplementary file 1 — Supplementary Figures and Materials and Tables [file 41388_2019_1050_MOESM1_ESM.doc]

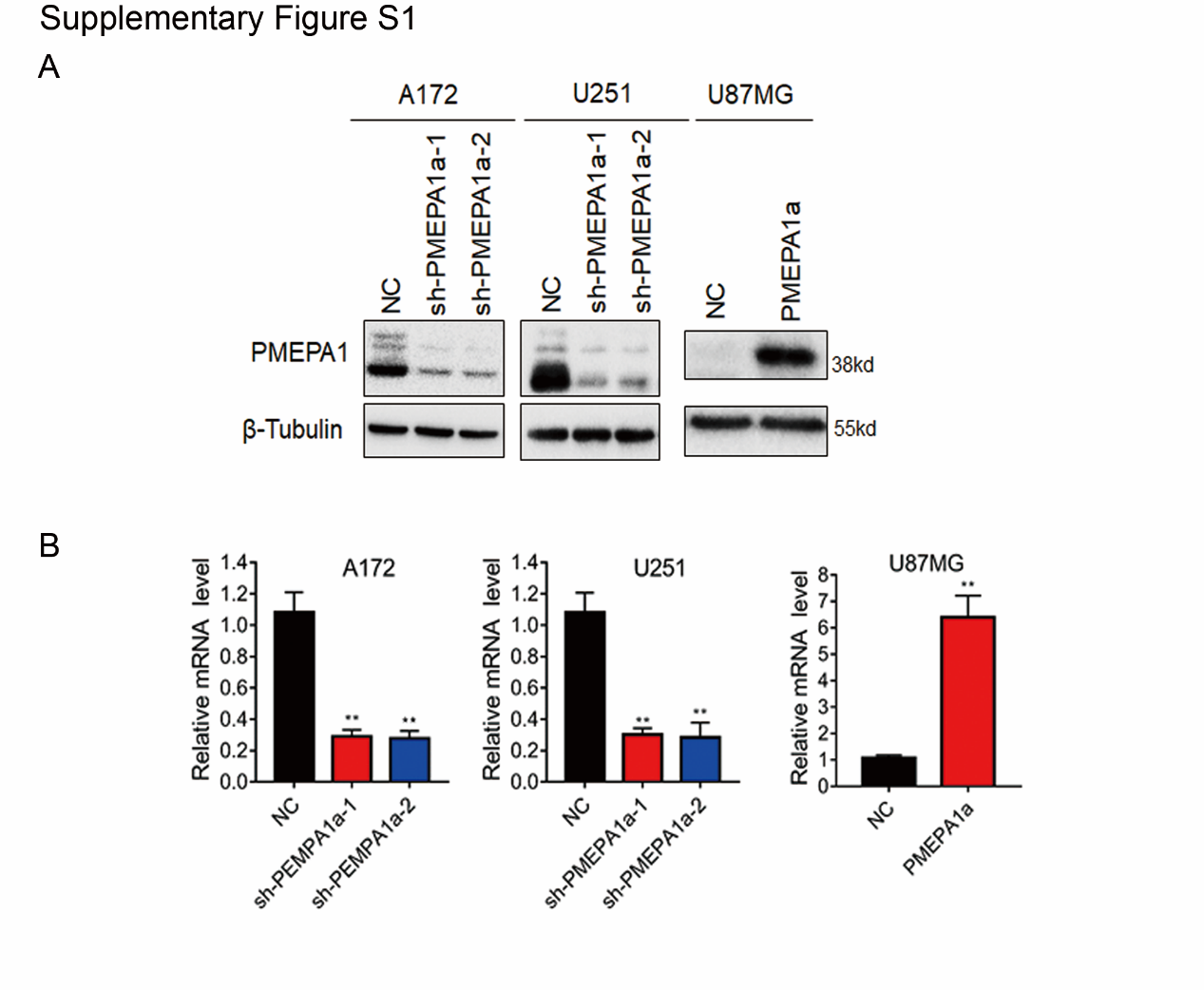


**Supplementary Figure S1**

A172 and U251 cells were infected with PMEPA1a shRNAs, U87MG cells were infected with PMEPA1a lentivirus. Knockdown or ectopic expression efficiency was confirmed by (A) western blotting and (B) qPCR. Data are represented as the mean ± SEM. Student’s *t*-test: ** *P* < 0.01.


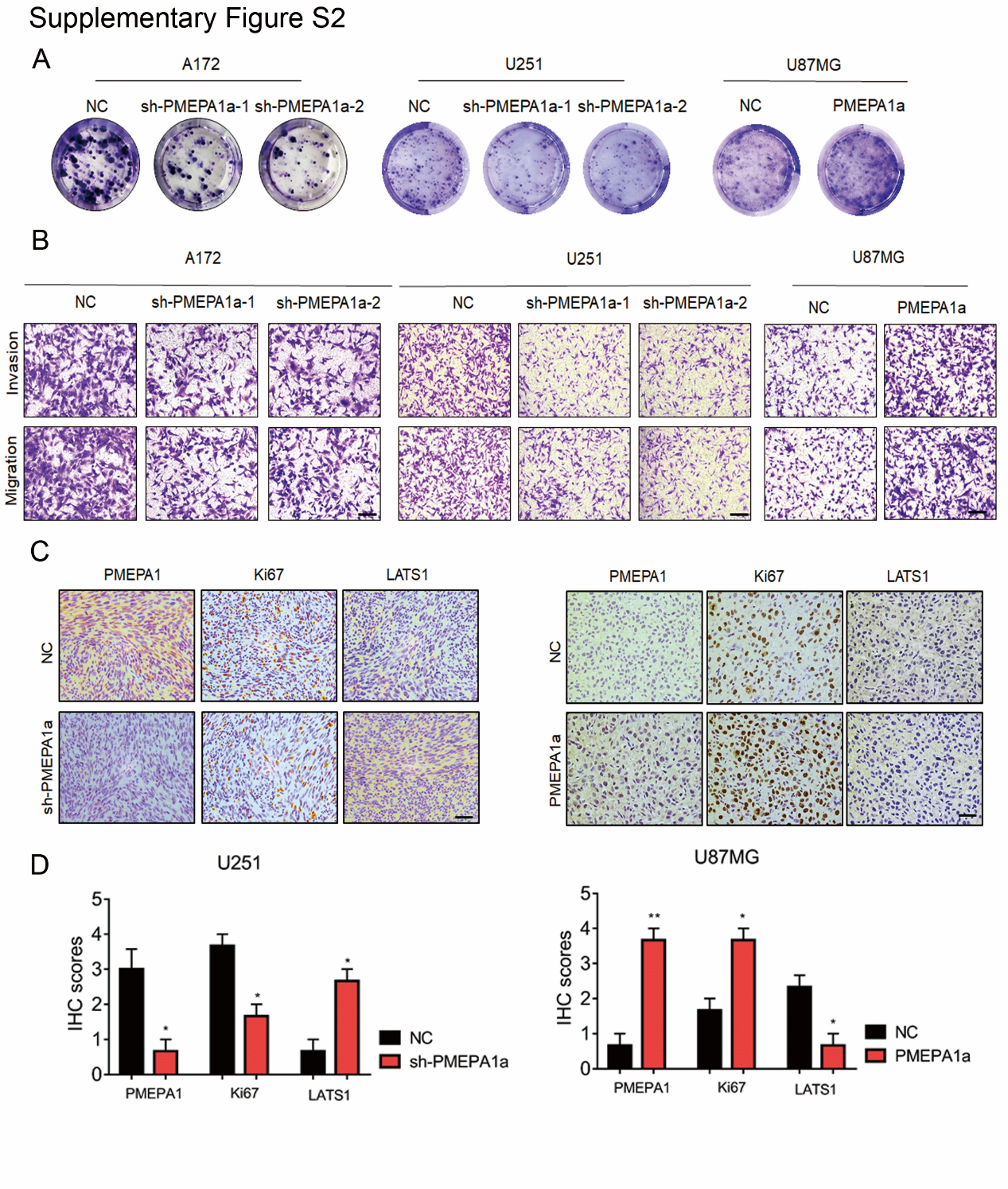


**Supplementary Figure S2**

(A, B) Representative images of colony forming assays and transwell assays performed on the indicated cells. Scale bars, 200 µm.

(C) Immunohistochemical staining for PMEPA1a, Ki67 and LATS1 in xenograft sections from U251 and U87MG groups. Scale bars, 50 µm.

(D) Graphic representation of IHC scoring of PMEPA1, Ki67, LATS1 levels in xenograft sections derived from U251 and U87MG groups. Data are represented as the mean ± SEM. Student’s *t*-test: n.s. = not significant, * *P* < 0.05, ** *P* < 0.01.


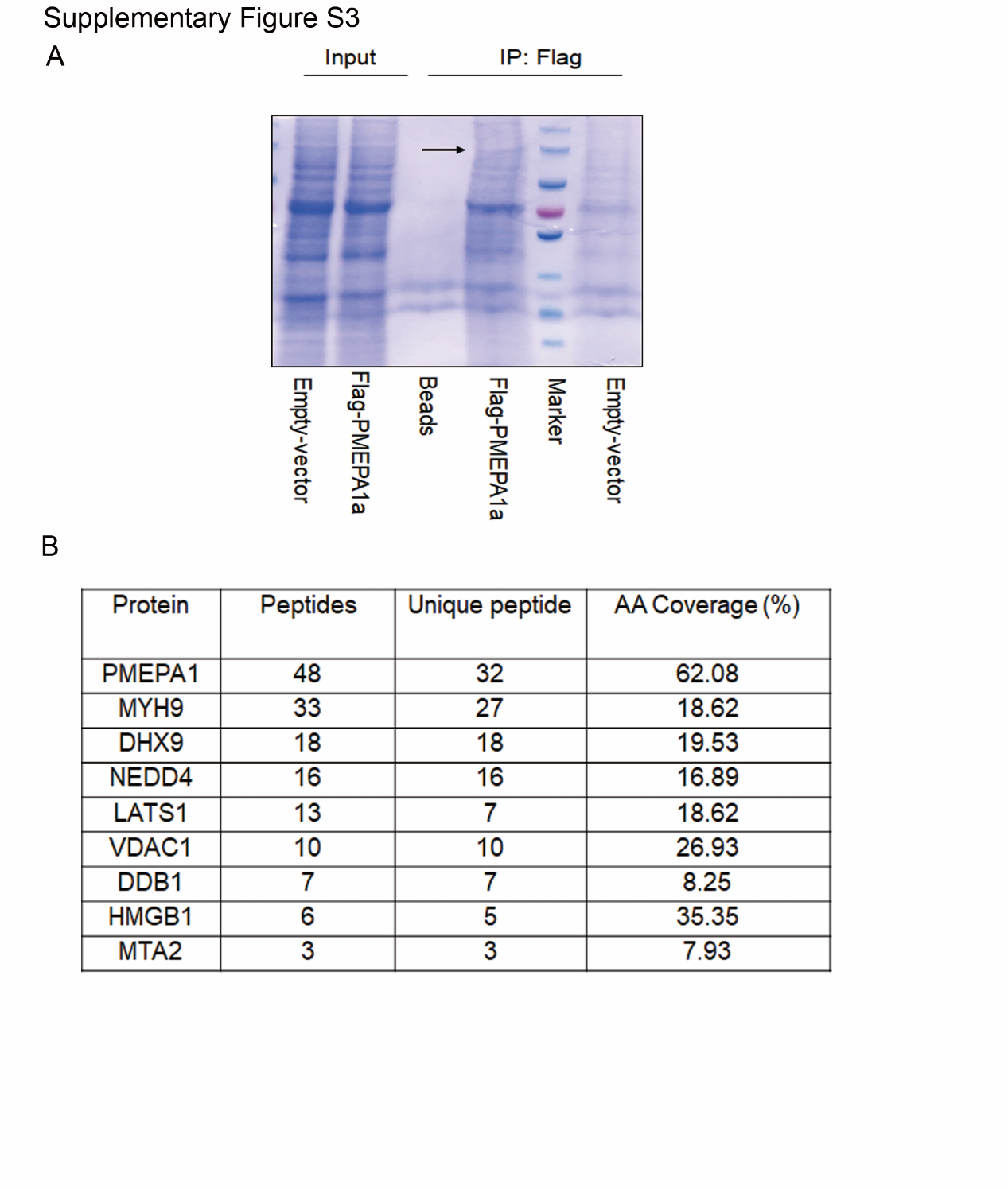


**Supplementary Figure S3**

1. Co-immunoprecipitations performed and analyzed in Commassie blue staining

(B) Mass spectrometry analysis of the proteins pulled down by anti-Flag in cells expressing Flag-PMEPA1a.


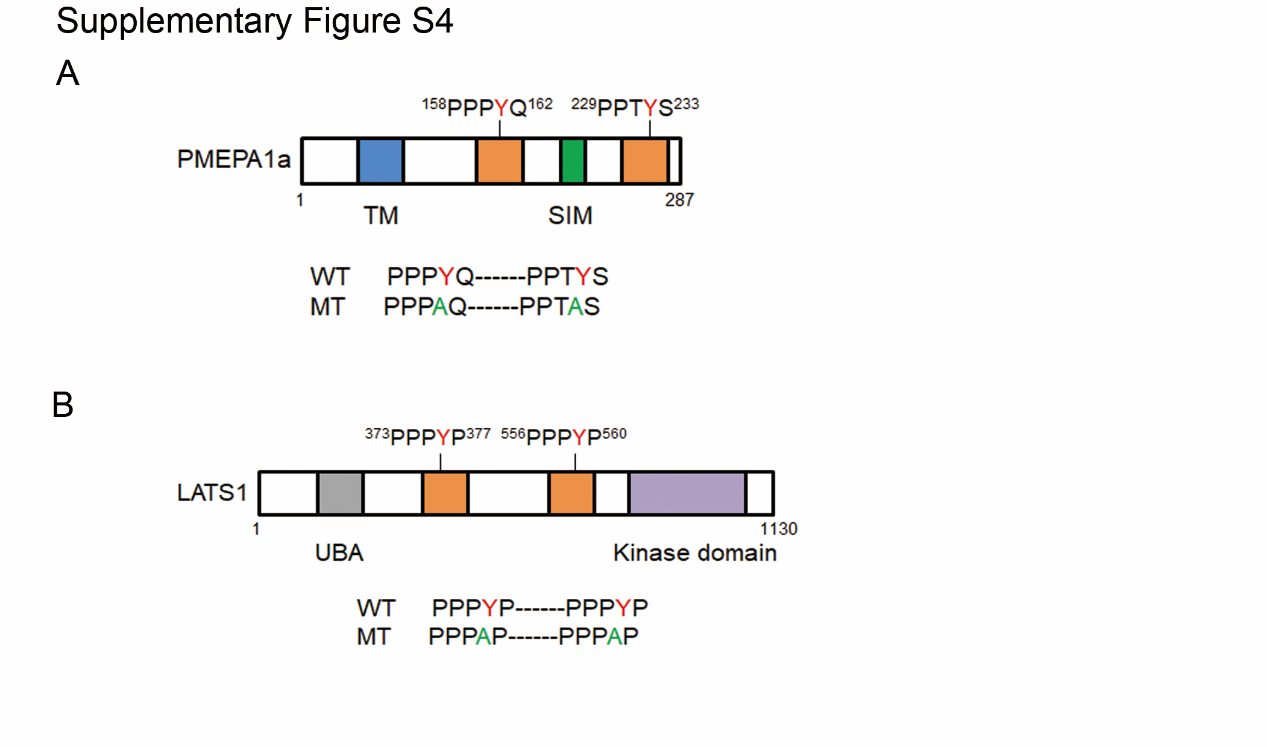


**Supplementary Figure S4**

(A) Schematic representation of wild-type PMEPA1a and mutated construct.

(B) Schematic representation of wild-type LATS1 and mutated construct.


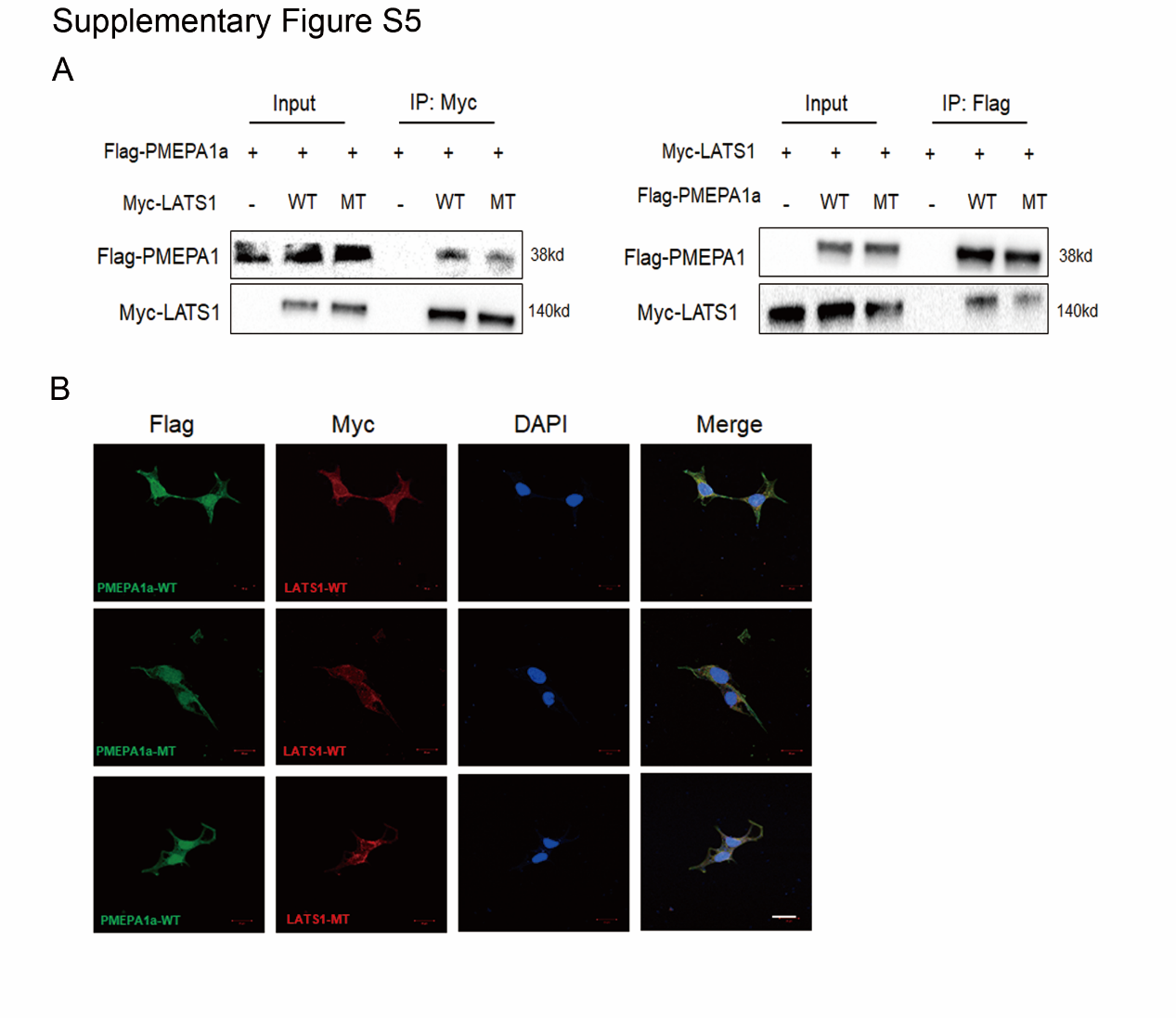


**Supplementary Figure S5**

(A) Western blot analysis of co-immunoprecipitations performed with lysates prepared from HEK293 cells transfected with Myc-LATS1 alone, or together with Flag-PMEPA1a (WT) or Flag-PMEPA1a (MT); or HEK293 cells transfected with Flag-PMEPA1a alone, or together with Myc-LATS1 (WT) or Myc-LATS1 (MT). Left side of panel includes total protein; right side includes Co-IPs. (B) Representative images from immunofluorescence staining, to demonstrate localization of PMEPA1a (green) and LATS1 (red), performed on HEK293 cells transfected with Myc-LATS1 (WT) and Flag-PMEPA1a (WT), or Myc-LATS1 (WT) and Flag-PMEPA1a (MT), or Myc-LATS1 (MT) and Flag-PMEPA1a (WT). Nuclei are stained with DAPI (blue). Images were acquired under confocal microscopy. Scale bars, 20 µm.


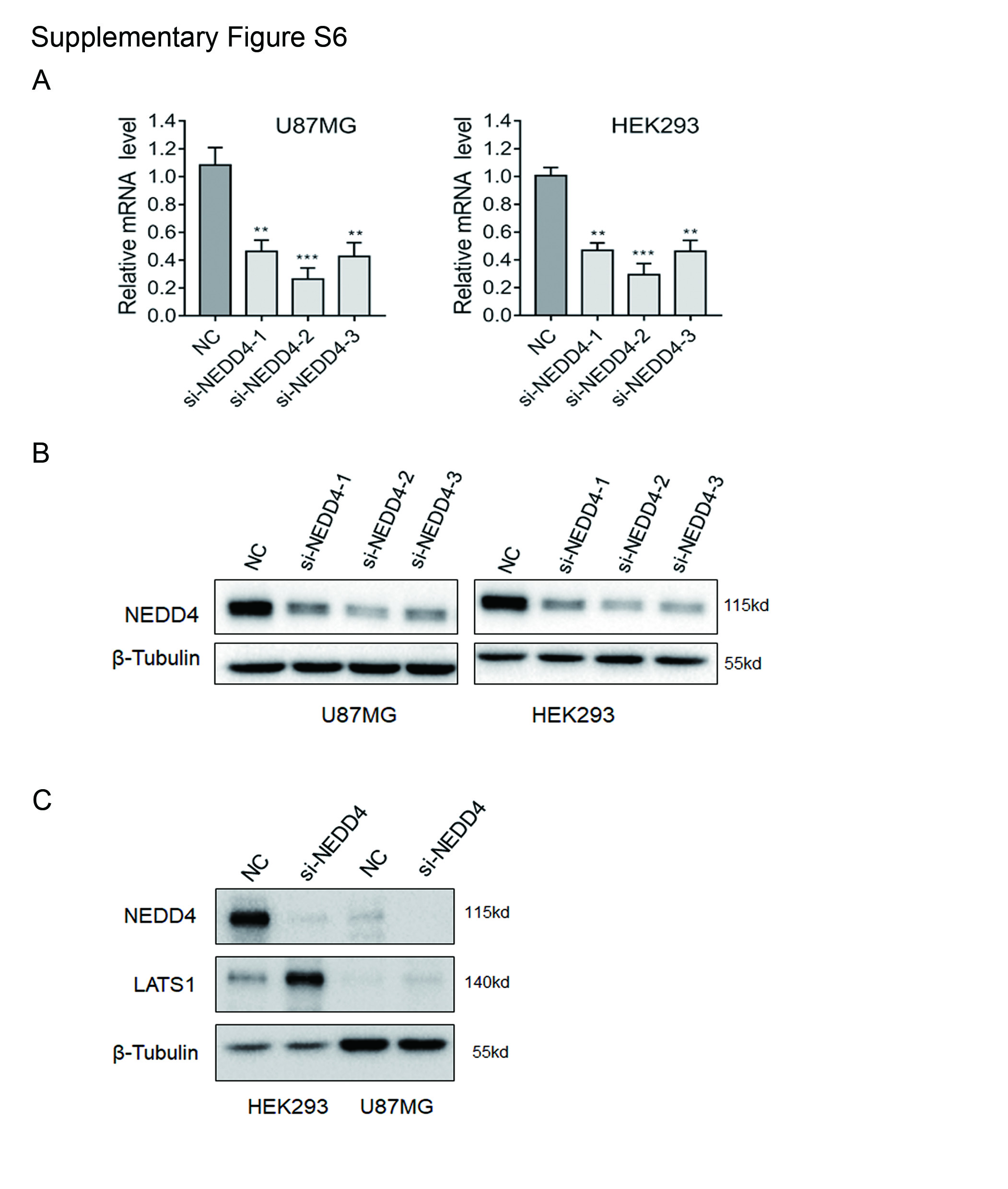


**Supplementary Figure S6**

U87MG and HEK293 cells were transfected with three siRNA targeting NEDD4, and knockdown efficiency was confirmed in (A) by qRT-PCR and (B) by western blot analysis. (C) Western blot analysis of lysates prepared from HEK293 and U87MG cells transfected with si-NEDD4. β-tubulin was used as loading control. Data are represented as the mean ± SEM. Student’s *t*-test: n.s. = not significant, ** *P* < 0.01, *** *P* < 0.001.


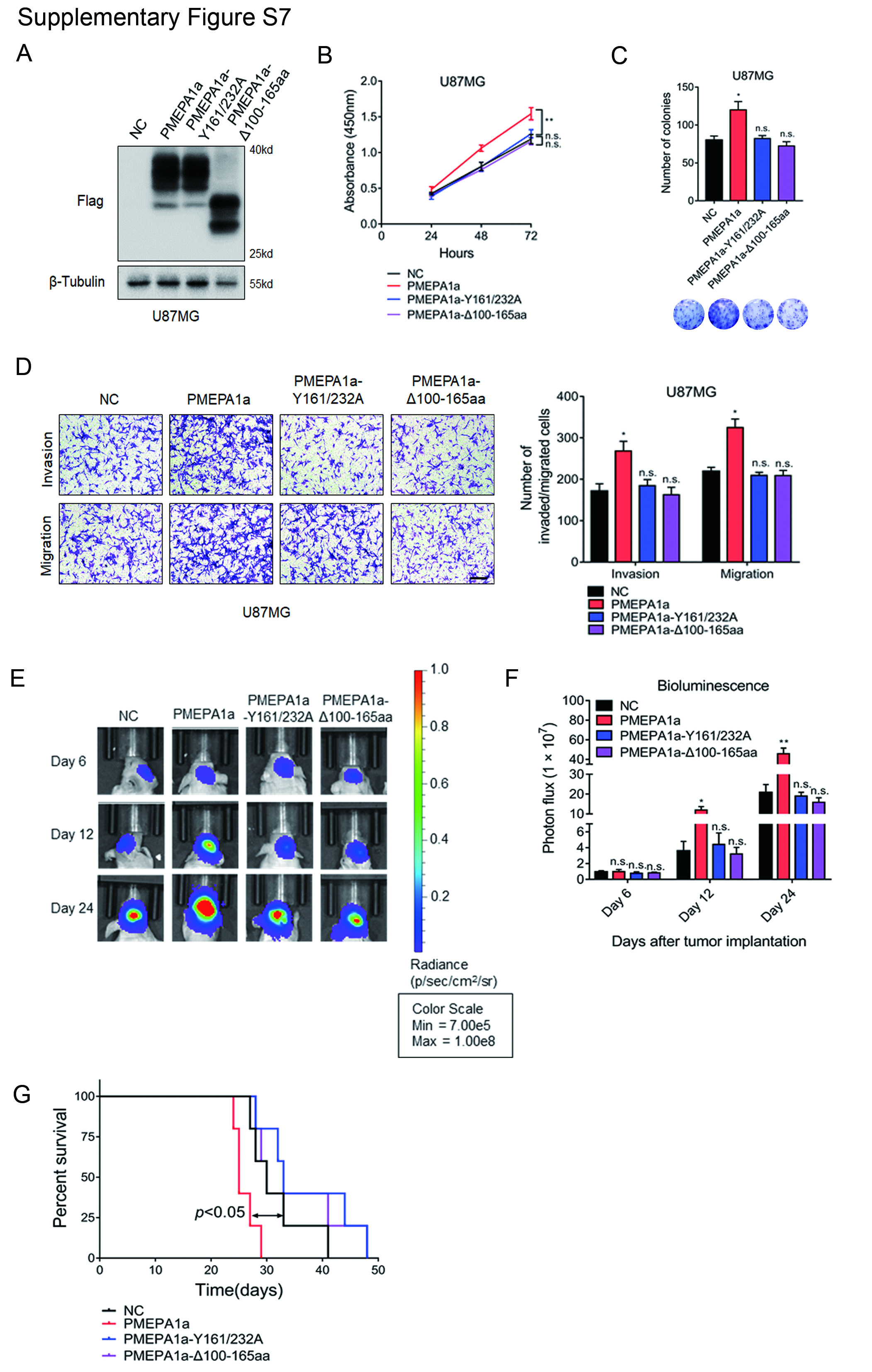


**Supplementary Figure S7**

(A) Western blot analysis of lysates prepared from U87MG cells infected with NC (control vector), Flag-PMEPA1a, Flag-PMEPA1a-Y161/232a or Flag-PMEPA1a-Δ100-165aa lentivirus. β-tubulin was used as loading control. (B) Absorbance values (450 nm) obtained from the CCK8 assay performed on modified U87MG cells as indicated. Data are represented as the mean ± SEM. (C) Representative images and graphic representation of the number of colonies in colony forming assay performed on modified U87MG cells as indicated. Data are represented as the mean ± SEM. (D) Representative images and graphic representation of the number of migrating cells in transwell assays performed on modified U87MG cells as indicated. Data are represented as the mean ± SEM. (E and F) *In vivo* bioluminescent images and quantification of U87MG-NC, -PMEPA1a, -PMEPA1a-Y161/232A, and -PMEPA1a-Δ100-165aa derived xenografts at the indicated time points. (F) Kaplan–Meier survival analysis performed with survival data from mice implanted with U87MG-NC, -PMEPA1a, -PMEPA1a-Y161/232A, and -PMEPA1a-Δ100-165aa cells. Log-rank test, PMEPA1a *vs* NC: *P* < 0.05; PMEPA1a-Y161/232A or -PMEPA1a-Δ100-165aa *vs* NC: *P* = n.s. Student’s t-test: n.s. = not significant, **P* < 0.05, ** *P* < 0.01.


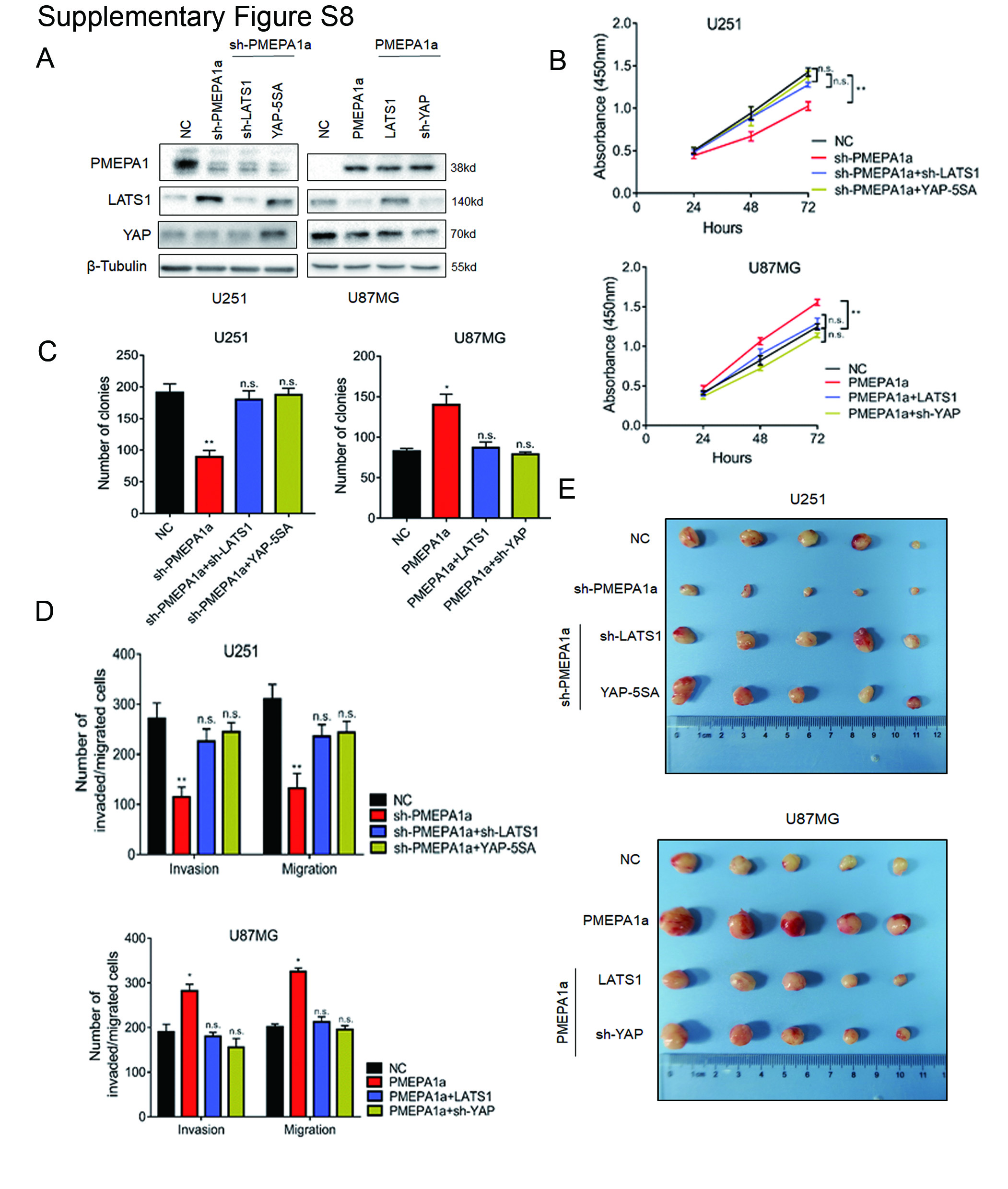


**Supplementary Figure S8. The Hippo pathway mediates oncogenic functions of PMEPA1a.**

(A) Western blot analysis of lysates prepared from U251 cells transduced with NC (control vector), sh-PMEPA1a, sh-LATS1 or YAP-5SA (constitutively active YAP), or U87MG cells transduced with NC, LATS1 or sh-YAP. β-tubulin was used as loading control. (B) Absorbance values (450 nm) obtained from the CCK8 assay performed on modified U251 and U87MG cells as indicated. Data are represented as the mean ± SEM. (C) Graphic representation of the number of colonies in colony forming assay performed on modified U251 and U87MG cells as indicated. Data are represented as the mean ± SEM. (D) Graphic representation of the number of migrating cells in transwell assays performed on modified U251 and U87MG cells as indicated. Data are represented as the mean ± SEM. (E) Representative images of subcutaneous xenografts from modified U251 and U87MG cells as indicated. Student’s t-test: n.s. = not significant, **P* < 0.05, ** *P* < 0.01.


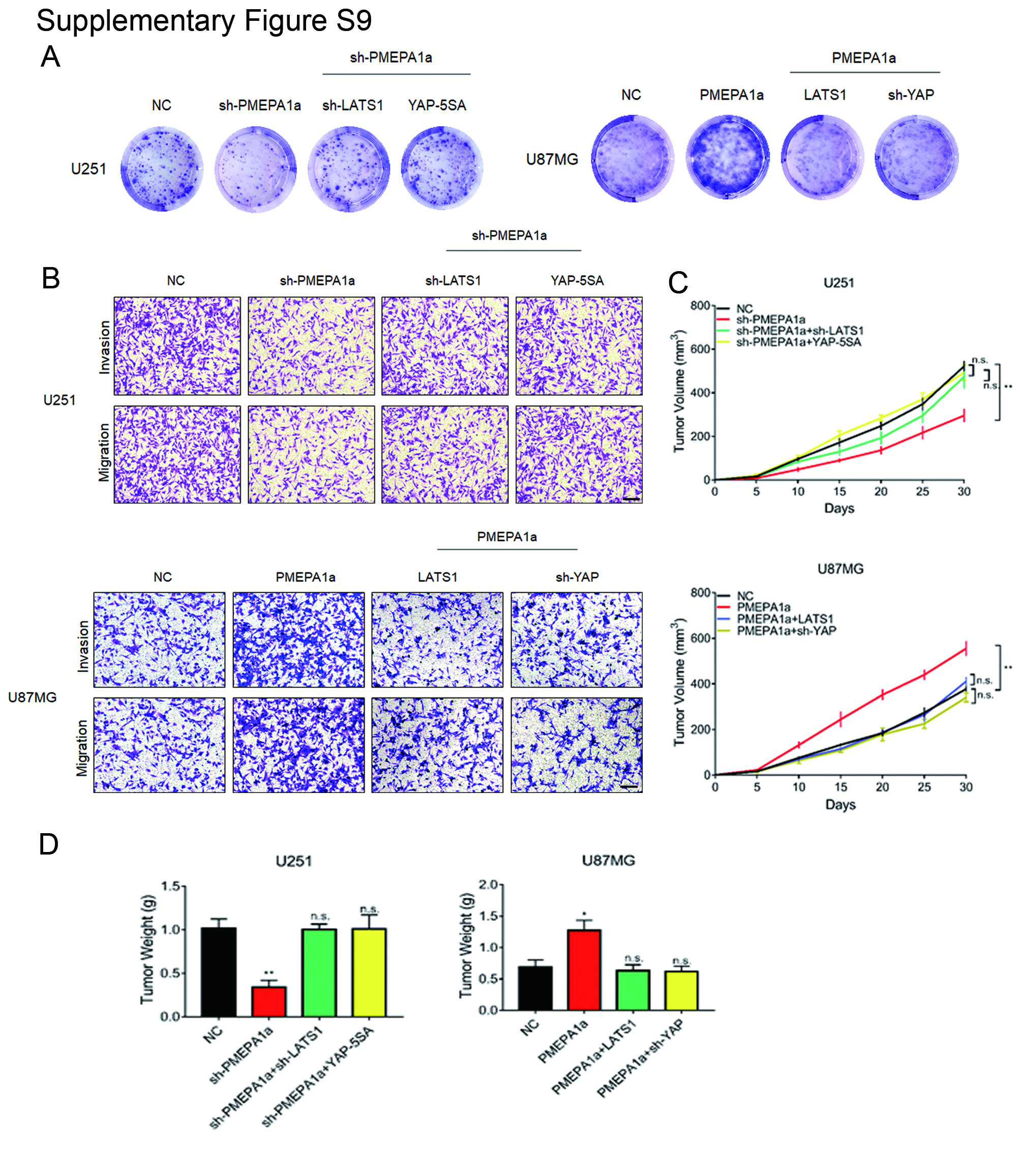


**Supplementary Figure S9**

(A) Representative images of colony forming assays performed on U251 or U87MG glioma cell lines modified for expression of PMEPA1a and Hippo signaling components LATS1 or YAP.

(B) Representative images of transwell assays performed on U251 or U87MG glioma cell lines modified for expression of PMEPA1a and Hippo signaling components LATS1 or YAP. Scale bar, 200 μm.

(C) Growth curves of subcutaneous xenografts in nude mice derived from the indicated cell types.

(D) Weight of subcutaneous xenografts from nude mice derived from indicated cell types. Student’s t-test: n.s. = not significant, **P* < 0.05, ***P* < 0.01.

**Supplementary Materials and Methods**

**Cell culture**

GBM, HEK293 and NHA cells were cultured in Dulbecco’s modified Eagle’s medium (DMEM; Thermo Fisher Scientific; Waltham, MA, USA) supplemented with 10% fetal bovine serum (FBS; Thermo Fisher Scientific). GBM#P3 cells were cultured in serum-free DMEM/F12 medium (Thermo Fisher Scientific) supplemented with 2% B27 Neuro Mix (Thermo Fisher Scientific), epidermal growth factor (20 ng/mL; Thermo Fisher Scientific), and basic fibroblast growth factor (10 ng/mL; Thermo Fisher Scientific).

**Transfection**

Stable knockdown of PMEPA1a was established in U251 and A172 cell lines using lentivirus containing two different short hairpin RNAs (shRNA) targeting PMEPA1a (U251- and A172-shRNA; OBiO Technology; Shanghai, China). Stable overexpression of PMEPA1a was established in U87MG cells infected with lentivirus expressing Flag-tagged full-length *PMEPA1a*, *PMEPA1a*-Δ100-165aa and *PMEPA1a*-Y161/232A (U87MG-PMEPA1a, -PMEPA1a-Δ100-165aa, -PMEPA1a-Y161/232A; OBiO Technology).

**Nuclear fractionation**

Cells were harvest and then centrifuge at 1000 × rpm for 5 minutes. After carefully removing and discarding the supernatant, cell pellet was incubated with ice-cold CER I for 10 minutes. Then cell pellet was treated with CER II for 1 minute. After being vortexed for 5 seconds on the highest setting, cell pellet was centrifuged for 5minutes at 13000 × g in a microcentrifuge. The supernatant (cytoplasmic extract) was transferred and stored in -80℃ and the rest insoluble pellet was treated with NER. The sample was vortexed for 15 seconds every 10 minutes, for a total of 4 times. After being centrifuged at 13000 × g for 10 minutes, the supernatant (nuclear extract) fraction was transferred to a clean tube and stored in -80℃ until use.

**Animal studies**

Animals were randomly divided into indicated groups. Animals were sacrificed by cervical dislocation if they showed any symptoms of discomfort, such as severe hunchback posture, decreased motion or activity, apathy, dragging legs, or more than 20% body weight loss. The brains were removed and fixed in 4% Paraformaldehyde (PFA), then sectioned and underwent hematoxylin-eosin (HE) and IHC staining.

**Antibodies used**

For western blotting, the following antibodies were used:, PMEPA1 (2A12, Novus Biologicals, lnc.; Littleton, CO; 1:1000); Histone H3 (ab176842, Abcam; Cambridge, MA; 1:1000), YAP (ab52771, Abcam; 1:1000), GAPDH (ab181602, Abcam; 1:10000), p-YAP (ab76252, Abcam; 1:1000); β-Tubulin (#2128, Cell Signaling Technology; Beverly, MA; 1:1000), CYR61(#14479, Cell Signaling Technology; 1:1000), p-LATS1 (#8654, Cell Signaling Technology; 1:1000), CTGF (#86641, Cell Signaling Technology; 1:1000), Myc-tag (#2276, Cell Signaling Technology; 1:1000), Flag-tag (#14793, Cell Signaling Technology; 1:1000), LATS1 (#3477, Cell Signaling Technology; 1:1000), HA-tag (#3724, Cell Signaling Technology; 1:1000), NEDD4 (#5344, Cell Signaling Technology; 1:1000); anti-IDH1 (R132H) (clone HMab-1, Sigma-Aldrich; St. Louis, MO; 1:1000), Flag-tag (F1804, Sigma-Aldrich; St. Louis, MO; 1:1000); Ub (sc-8017, Santa Cruz Biotechnology; Dallas, TX; 1:200).

Anti-YAP (ab52771, Abcam; 1:200) was used in immunofluorescence assays.

For immunohistochemistry, anti-PMEPA1 (2A12, Novus Biologicals, lnc.), anti-LATS1 (#3477, Cell Signaling Technology), anti-p-YAP (ab76252, Abcam) and anti-CYR61 (#14479, Cell Signaling Technology) were used at a dilution of 1:200.

Co-IPs were performed with non-specific IgG or antibodies specific for LATS1 (#3477, Cell Signaling Technology; 4 µL), PMEPA1 (2A12, Novus Biologicals, lnc.; 4 µL); HA-tag (#3724, Cell Signaling Technology; 4 µL), and Flag-tag (F1804, Sigma-Aldrich; 4 µL).

**Supplementary Table S1. Association between PMEPA1 expression and clinicopathologic factors in glioma patients**

| Variables | Cases (*n*) | *PMEPA1* expression  low high | | *P*-value |
| --- | --- | --- | --- | --- |
| Age (years) |  |  |  |  |
| < 60 | 33 | 17 | 16 | 0.7550 |
| ≥ 60 | 27 | 15 | 12 |
| Gender |  |  |  |  |
| Male | 39 | 21 | 18 | 0.9136 |
| Female | 21 | 11 | 10 |
| Tumor size (cm) |  |  |  |  |
| < 4 | 37 | 20 | 17 | 0.8871 |
| ≥ 4 | 23 | 12 | 11 |
| Cystic change |  |  |  |  |
| Absent | 22 | 10 | 12 | 0.3520 |
| Present | 38 | 22 | 16 |
| Edema |  |  |  |  |
| None to mild | 43 | 22 | 21 | 0.5920 |
| Moderate to severe | 17 | 10 | 7 |
| WHO grade |  |  |  |  |
| LGG | 20 | 16 | 4 | 0.0034 |
| HGG | 40 | 16 | 24 |

LGG: Low grade glioma; HGG: High grade glioma.

**Supplementary Table S2. Plasmids used in this study**

| **Plasmids** | **Resources** |
| --- | --- |
| pGL3-Control Vector | Promega |
| pGL4-SV40 Driven Renilla Luciferase Vector | Promega |
| 8xGTIIC-luciferase | Addgene |
| pcDNA3.1-3xFlag-empty vector | OBiO Technology |
| pcDNA3.1-PMEPA1a-wt-3xFlag | OBiO Technology |
| pcDNA3.1-PMEPA1a-2YA-3xFlag | OBiO Technology |
| pcDNA3.1-PMEPA1a-3xFlag (1-225aa) | OBiO Technology |
| pcDNA3.1-PMEPA1a-3xFlag (1-165aa) | OBiO Technology |
| pcDNA3.1-PMEPA1a-3xFlag (1-100aa) | OBiO Technology |
| pcDNA3.1-HA-empty vector | OBiO Technology |
| pcDNA3.1-HA-UBB | OBiO Technology |
| pcDNA3.1-Myc-empty vector | OBiO Technology |
| pcDNA3.1-Myc-LATS1 | OBiO Technology |
| pcDNA3.1-Myc-LATS1-2YA  pcDNA3.1-Myc-LATS1 (1-350aa)  pcDNA3.1-Myc-LATS1 (351-700aa)  pcDNA3.1-Myc-LATS1 (701-1130aa) | OBiO Technology  OBiO Technology  OBiO Technology  OBiO Technology |

**Supplementary Table S3. Oligonucleotide sets used in this study**

| **siRNAs or shRNAs** | **Sequences** |
| --- | --- |
| siNEDD4-1 | 5'-GCACUAGUGCUAAAGGAUUTT-3' |
| siNEDD4-2 | 5'-GCAUCGAGCUCAAAUCAUUTT-3' |
| siNEDD4-3 | 5'-CCAAUGAUCUAGGGCCUUUTT-3' |
| si-NC | 5'-UUCUCCGAACGUGUCACGUTT-3' |
| sh-PMEPA1a-1 | 5'-GCAACTGCAAACGCTCTTT-3' |
| sh-PMEPA1a-2 | 5'-GCAAACGCTCTTTGTTCCA-3' |
| sh-YAP | 5'-CCCAGTTAAATGTTCACCAAT-3' |
| sh-LATS1 | 5'-GCAGACAACCAATCATCAT-3' |
| sh-NC | 5'-TTCTCCGAAGGTGTCACGG-3' |

**Supplementary Table S4 Primer sets used in this study**

| **Primer set** | **Primers** | **Sequence (5’-3’)** | **Product size (bp)** |
| --- | --- | --- | --- |
| PMEPA1-a | F | 5'-CTGCAAACGCTCTTTGTTCCA-3' | 133 |
|  | R | 5'-TGCAGACAGCTTGTAGTGGC -3' |  |
| PMEPA1-b | F | 5'-ACGCGAGTTCCCGTCTTTC -3' | 147 |
|  | R | 5'-AAACAAACTCCAGCTCCGCCA-3' |  |
| PMEPA1-c | F | 5'-GCGAGTTCCCGTCTTTCCTG-3' | 140 |
|  | R | 5'-TGAACAAACTCCAGCTCCGCTG-3' |  |
| PMEPA1-d | F | 5'-GACCCTCTACACCCGCCATA-3' | 180 |
|  | R | 5'-GGCATTTTGACTTTTCGCCTG-3' |  |
| GAPDH | F | 5'-GCACCGTCAAGGCTGAGAAC-3' | 138 |
|  | R | 5'-TGGTGAAGACGCCAGTGGA-3' |  |
| LATS1 | F | 5'-CTCTTCACATTCCACCACAAGC-3' | 88 |
|  | R | 5'-CCTAAGCGATCTTCGGGTCC-3' |  |
| YAP1 | F | 5'-TGACCCTCGTTTTGCCATGA-3' | 125 |
|  | R | 5'-GTTGCTGCTGGTTGGAGTTG-3' |  |
| CTGF | F | 5'-TGGAAGAGAACATTAAGAAGGGCA -3' | 84 |
|  | R | 5'-TGCAGCCAGAAAGCTCAAAC-3' |  |
| CYR61 | F | 5'-GCAAGGAGCTGGGATTCGAT-3' | 106 |
|  | R | 5'-ATTCCAAAAACAGGGAGCCG-3' |  |
| ANKRD1 | F | 5'-GCAAAAATTAGCGCCCGAGA-3' | 139 |
|  | R | 5'-ACGGGGTATCTCCTTCTCTGT-3' |  |
| BIRC5 | F | 5'-TGACGACCCCATAGAGGAACA-3' | 186 |
|  | R | 5'-CGCACTTTCTCCGCAGTTTC-3' |  |
| DIAPH3  NEDD4 | F | 5'-CCTACCCTTCCTCAGCCTC-3' | 162 |
|  | R  F  R | 5'-TGTCCAGCATATCATCCGTCAG-3'  5'-TCTAAAGGGGGACCATCTCAGT-3'  5'-CCTAGGAAATCATCTCTTGTCTTGC-3' | 140 |
